# Maternal treatment with short-chain fatty acids modulates the intestinal microbiota and immunity and ameliorates type 1 diabetes in the offspring

**DOI:** 10.1371/journal.pone.0183786

**Published:** 2017-09-08

**Authors:** James C. Needell, Diana Ir, Charles E. Robertson, Miranda E. Kroehl, Daniel N. Frank, Danny Zipris

**Affiliations:** 1 Barbara Davis Center for Childhood Diabetes, University of Colorado Denver, Aurora, Colorado, United States of America; 2 Division of Infectious Diseases, University of Colorado School of Medicine, Aurora, Colorado, United States of America; 3 University of Colorado Microbiome Research Consortium (MiRC), Aurora, Colorado, United States of America; 4 Department of Biostatistics and Informatics, Colorado School of Public Health and University of Colorado Denver, Aurora, Colorado, United States of America; Universite du Quebec a Montreal, CANADA

## Abstract

We recently hypothesized that the intestinal microbiota and the innate immune system play key roles in the mechanism of Kilham Rat Virus-induced type 1 diabetes in the LEW1.WR1 rat. We used this animal model to test the hypothesis that maternal therapy with short-chain fatty acids can modulate the intestinal microbiota and reverse virus-induced proinflammatory responses and type 1 diabetes in rat offspring. We observed that administration of short-chain fatty acids to rat breeders via drinking water prior to pregnancy and further treatment of the offspring with short-chain fatty acids after weaning led to disease amelioration. In contrast, rats that were administered short-chain fatty acids beginning at weaning were not protected from type 1 diabetes. Short-chain fatty acid therapy exerted a profound effect on the intestinal microbiome in the offspring reflected by a reduction and an increase in the abundances of Firmicutes and Bacteroidetes taxa, respectively, on day 5 post-infection, and reversed virus-induced alterations in certain bacterial taxa. Principal component analysis and permutation multivariate analysis of variance tests further revealed that short-chain fatty acids induce a distinct intestinal microbiota compared with uninfected animals or rats that receive the virus only. Short-chain fatty acids downregulated Kilham Rat Virus-induced proinflammatory responses in the intestine. Finally, short-chain fatty acids altered the B and T cell compartments in Peyer’s patches. These data demonstrate that short-chain fatty acids can reshape the intestinal microbiota and prevent virus-induced islet autoimmunity and may therefore represent a useful therapeutic strategy for disease prevention.

## Introduction

The intestinal microbiota is critical for gut development, metabolism, and normal immune function (reviewed in refs. [1, 2]). Emerging data from both humans and animals have implicated alterations in gut bacterial composition (“dysbiosis”) in the pathogenicity of several proinflammatory disorders, including rheumatoid arthritis, inflammatory bowel disease, and metabolic syndrome (reviewed in ref. [3]).

Evidence from animal models of type 1 diabetes (T1D) suggests that dysbiosis may be among the mechanisms associated with disease progression [4–6]. Furthermore, recent studies have implied that altered gut bacterial composition may be linked with the development of T1D in humans [7–9]. Whether these changes in the gut microbiome are directly linked with disease mechanisms remains to be determined [10, 11]. It is hypothesized that the dietary intake in developed nations has shifted to a high-fat, high carbohydrate, low-fiber diet that may have resulted in functional changes in the intestinal microbiota [12–15]. Such diet-induced alterations to gut bacterial communities are now postulated to play a key role in the rising incidence of proinflammatory disorders in the developed world, including obesity, inflammatory bowel disease, and T1D [16]. Short-chain fatty acids (SCFAs) produced by the fermentation of indigestible dietary plant fiber by the intestinal microbiota (reviewed in refs. [17, 18]) can enter the circulation and regulate innate and adaptive immunity [19].

Formate (C_1_), acetate (C2), propionate (C_3_) and butyrate (C_4_) are short chain fatty acids (SCFAs) found in the intestine at concentrations of approximately 13 mM in the terminal ileum, ~130 mM in the caecum and ~80 mM in the descending colon and are generated by anaerobic bacterial fermentation of non-digestible plant fiber [20–22]. SCFAs released in the intestine have been linked with modulation of the immune system in and outside the digestive tract [22, 23]. Short-chain fatty acids enter the circulation [13] and can modulate the production of proinflammatory cytokines such as TNFα, IL-6, and IFN-γ via mechanisms hypothesized to involve downregulation of histone deacetylase activity [22] and the activation of specific receptors such as G protein–coupled receptor 43 (GPR43) [22]. A number of examined the effect of SCFAs and their derivatives on proinflammatory disorders in humans and animal models and observed beneficial effects in inflammatory bowel disease, sepsis, and ischemia induced injury [22].

The infection of LEW1.WR1 rats with Kilham Rat Virus (KRV) results in beta cell inflammation and T1D via mechanisms that may involve alteration of the intestinal microbiota [5] and upregulation of the innate immune system [24]. Due to the anti-inflammatory properties of SCFAs [17], we sought to determine whether these substances could modulate the gut bacterial composition and KRV-induced inflammation and prevent islet autoimmunity. We found that SCFA therapy that commences prenatally but not after weaning can alter the intestinal microbiota and virus-induced intestinal immunity and ameliorate diabetes in LEW1.WR1 offspring.

## Material and methods

### Animals and viruses

Specific pathogen-free LEW1.WR1 rats of both sexes were obtained from BRM Inc. (Worcester, MA) and were bred and housed in our specific pathogen-free facility. The study was carried out in strict accordance with the recommendations in the Guide for the Care and Use of Laboratory Animals of the National Institutes of Health. The protocol was approved by the Committee on the Ethics of Animal Experiments of the University of Colorado Denver (Permit Number: B-79715(10)1E). KRV was propagated and titered as previously described [25].

### Histological staining

Pancreatic tissue was fixed for 24 h in 10% neutral-buffered formalin, embedded in paraffin, cut (5–6 μm slices), and mounted on microscope slides. The tissue was stained with hematoxylin and eosin.

### Virus-induced diabetes, SCFA treatments, and blood and lymphoid organ removal

**Fig 1:**
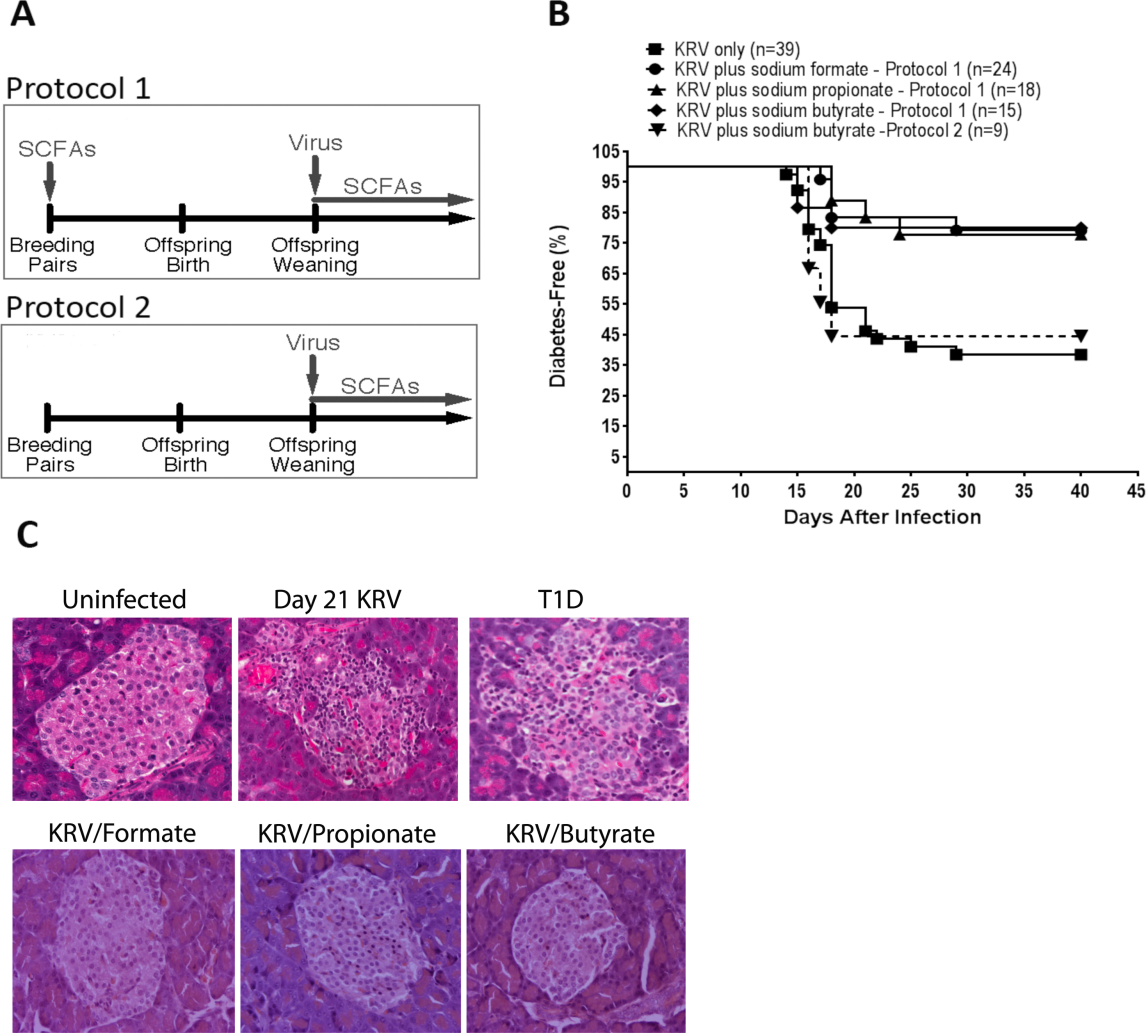
Kaplan-Meier analysis of virus-induced T1D in LEW1.WR1 rats treated with SCFAs. LEW1.WR1 rat breeders were treated with 250 mM of the various SCFAs beginning at the age of 6 weeks prior to any pregnancy (Protocol 1). The offspring were injected with 1 x 10^7^ PFU of KRV at 21–25 days of age and continued to receive SCFAs after weaning for 40 days following virus inoculation as shown in Fig 1A. A second animal group was injected with KRV and treated with 250 mM of sodium butyrate in the drinking water beginning at weaning (Protocol 2). Another group was administered with KRV only. Diabetes was defined as the presence of plasma glucose concentrations >250 mg/dl (11.1 mmol/L) on 2 consecutive days. Survival was analyzed using the Kaplan-Meier method as shown in Fig 1B. Statistical analyses among groups were performed using the log rank test (*p* = 0.001 for formate; *p* = 0.005 for propionate; and *p* = 0.006 for butyrate versus KRV only). Shown are paraffin sections of hematoxylin- and eosin-stained sections of pancreatic tissue isolated from uninfected control rats, KRV-infected rats at 21 days following infection (insulitis), KRV- plus SCFA-treated animals at 40 days following infection as indicated, and KRV-infected rats at diabetes onset (Panel C).

Rats at 21–25 days of age were either left untreated or injected i.p. with 1 x 10^7^ PFU of KRV and monitored for diabetes for 40 days following virus inoculation, as previously described [26]. We analyzed the effect of butyric acid, the most studied SCFA (Alfa Aesar, Ward Hill, MA) versus that of sodium formate and sodium propionate (both from Sigma-Aldrich, Saint Louis, MI). SCFAs were dissolved in drinking water and changed once a week. To examine the effect of SCFA therapy on the intestinal microbiome and the development of virus-induced T1D, we used 2 treatment protocols, indicated in Fig 1A. In Protocol 1, rat breeders were fed for life with 250 mM of sodium formate, sodium propionate, or sodium butyrate via drinking water beginning at the age of 6 weeks, prior to any pregnancy. At 21–25 days of age, the offspring were weaned and infected with 1 x 10^7^ PFUs of KRV and continued to be fed 250 mM of sodium formate, sodium propionate, or sodium butyrate via drinking water until day 40 after KRV infection when all experiments were terminated. Control groups included rats treated with SCFAs only. In Protocol 2, a proof-of-principle study was carried out to examine the effect of feeding rats 250 mM of sodium butyrate beginning at the age of 21–25 days continuing until day 40. Peyer’s patches were collected 5 days after viral inoculation. Cells were obtained by passing the tissue through a 70 μm nylon mesh filter. The cells were washed and resuspended in tissue culture media or PBS for further use.

### High-throughput DNA sequencing and quantitative PCR analysis for microbiome analysis

Bacterial profiles were determined by broad-range amplification and sequence analysis of 16S rRNA genes following our previously described methods [4, 5, 8]. In brief, amplicons were generated using barcoded primers targeting the bacterial 16S rRNA V1V2 variable region [27]. PCR products were normalized using a SequalPrep kit (Invitrogen, Carlsbad, CA), pooled, lyophilized, purified and concentrated using a DNA Clean and Concentrator Kit (Zymo, Irvine, CA). Pooled amplicons were quantified using Qubit Fluorometer 2.0 (Invitrogen, Carlsbad, CA) and Illumina paired-end sequencing performed on the Miseq platform, using a 600-cycle version 3 reagent kit. Paired-end reads were aligned to Norwegian rat reference genome rn4 with bowtie2 and matching sequences discarded (ref. [28], and Homo Sapiens UCSC Hg19 Human Genome Sequence from iGenome [http://support.illumina.com/sequencing/sequencing_software/igenome.ilmn]]. The remaining non-host paired-end sequences were sorted by sample via barcodes in the paired reads with a python script [4]. The sorted paired reads were assembled using phrap [29] and pairs that did not assemble were discarded. Assembled sequence ends were trimmed over a moving window of 5 nucleotides until average quality met or exceeded 20. Trimmed sequences with more than 1 ambiguity or shorter than 250 nt were discarded. Potential chimeras identified with Uchime (usearch6.0.203_i86linux32) [30] using the Schloss [31] Silva reference sequences were removed from subsequent analyses. This process generated 15,034,637 high-quality 16S rRNA sequences for 43 samples (average sequence length: 297 nt; median sequence/sample: 339,580, range: 232,287 to 497,742). The median Goods coverage score was ≥ 99.994% at the rarefaction point of 237,113. The software package Explicet (v2.10.5) [32] was used for display and analysis.

For quantitative PCR analysis, bacterial DNA was recovered using the QIAmp DNA stool mini kit (Qiagen, Valencia, CA) according to the manufacturer’s instructions. DNA from *Lactobacillus*, *Bifidobacterium*, *Clostridium*, and *Bacteroides* was detected by quantitative PCR analysis using previously published primers [5]. The data were normalized to the total bacterial DNA in each sample using recently described primers and conditions [33].

### RNA extraction, cDNA synthesis, and quantitative RT-PCR

RNA extraction, cDNA synthesis and quantitative RT-PCR were performed as previously described [34]. A melting point analysis was performed in all cases to confirm the presence of the expected gene product. The standards used for the gene amplification were TOPO plasmid vectors (Invitrogen) expressing a ~500 bp DNA fragment derived from the mRNA sequence of the gene of interest that includes the ~100 bp sequence used for the PCR amplification. The primers were synthesized by Integrated DNA Technologies (IDT, Coralville, IA). Their sequences have been previously published [5, 34].

### Flow cytometry

Cells were resuspended in PBS containing 1% BSA and 0.1% sodium azide incubated with optimal concentrations of fluorochrome-conjugated Abs for 30 min at 4°C, washed, and fixed with 1% paraformaldehyde. A PE-conjugated anti-IL-2Rα-chain Ab (CD25, clone OX-39, mouse IgG1), an APC-conjugated anti-CD4 Ab (clone OX-35, mouse IgG2a), a PerCP-conjugated anti-CD8α chain Ab (clone OX-8, mouse IgG1), a FITC-conjugated anti-CD45R Ab (a marker of B cells, clone HIS24, mouse IgG2b), and appropriate isotype controls were purchased from BioLegend (San Diego, CA). An efluor 450-conjugated mAb against Foxp3 (Clone FJK-16s, rat IGg2a) and fixation and permeabilization buffers were purchased from eBioscience (San Diego, CA). Flow cytometry was performed using a CYAN ADP instrument (Beckman Coulter), and the results were analyzed with FlowJo software.

### Statistical analysis

Statistical comparisons of diabetes-free survival among groups were performed using the method of Kaplan and Meier. Data analysis was performed as we recently described [8]. For comparisons made across all groups, an overall Wilcoxon rank based test was used. If this overall *p-value* was significant (*p*<0.05) the pairwise differences were calculated. A false discovery rate (FDR) adjustment for multiple comparisons was used on the pairwise difference tests; comparisons with p<0.10 after FDR were considered significant. Differences in microbiome composition (i.e., beta-diversity) between subsets were quantified by the Bray-Curtis index using the *vegan* R package, which performs a non-parametric multivariate analysis of variance (PERMANOVA with 10,000 replicate resamplings) [35]. Data obtained from quantitative PCR, flow cytometry, and ELISA experiments were statistically analyzed using a one-way ANOVA with Bonferroni’s multiple comparison test as we recently published [5].

## Results

### Short chain fatty acids prevent virus-induced T1D in LEW1.WR1 rats

We tested the hypothesis that SCFA therapy that commences prenatally can ameliorate T1D. To this end, rat breeders at the age of 6 weeks were given 250 mM in drinking water as described in *Materials and Methods*. We preferred administering SCFAs via drinking water over oral gavage because the latter has been shown to induce significant stress and could therefore affect the results [36]. Animals treated with SCFAs did not show any signs of morbidity or mortality, altered growth, or abnormal behavior at any time point during the experiment. Compared with KRV infection without SCFA therapy, treating rats with sodium butyrate (n = 15), sodium formate (n = 24), or sodium propionate (n = 18) reduced the incidence of T1D to ~20% versus 60% (24/39) in animals that received KRV alone (*p* = 0.006 for butyrate; *p* = 0.001 for formate; *p* = 0.005 for propionate). Given this observation, we were interested to see whether SCFA therapy beginning at weaning could also prevent virus-induced T1D. The data presented in Fig 1B indicate that ~60% (5/9) of animals treated with KRV plus SCFAs beginning at weaning developed diabetes (*p* = 0.7 versus KRV only).

We next determined whether SCFA-induced disease prevention was associated with altered insulitis (*n* = 3–5 per group; ≥20 islets per animal were screened for insulitis). We observed that the pancreases from KRV-infected rats showed insulitis in the majority of the islets on day 21 following virus inoculation, with severe islet destruction observed following disease onset (Fig 1C). In contrast, the majority of the pancreatic tissue from rats infected with KRV and treated with SCFAs had only minimal or was insulitis-free (Fig 1C).

Together, these data imply that therapy with SCFAs interferes with the course of T1D and protects LEW1.WR1 rats from beta cell destruction. The data further underscore the importance the timing of the SCFA therapy in disease prevention.

### SCFA-treated LEW1.WR1 rats harbor altered gut microbiomes

**Fig 2:**
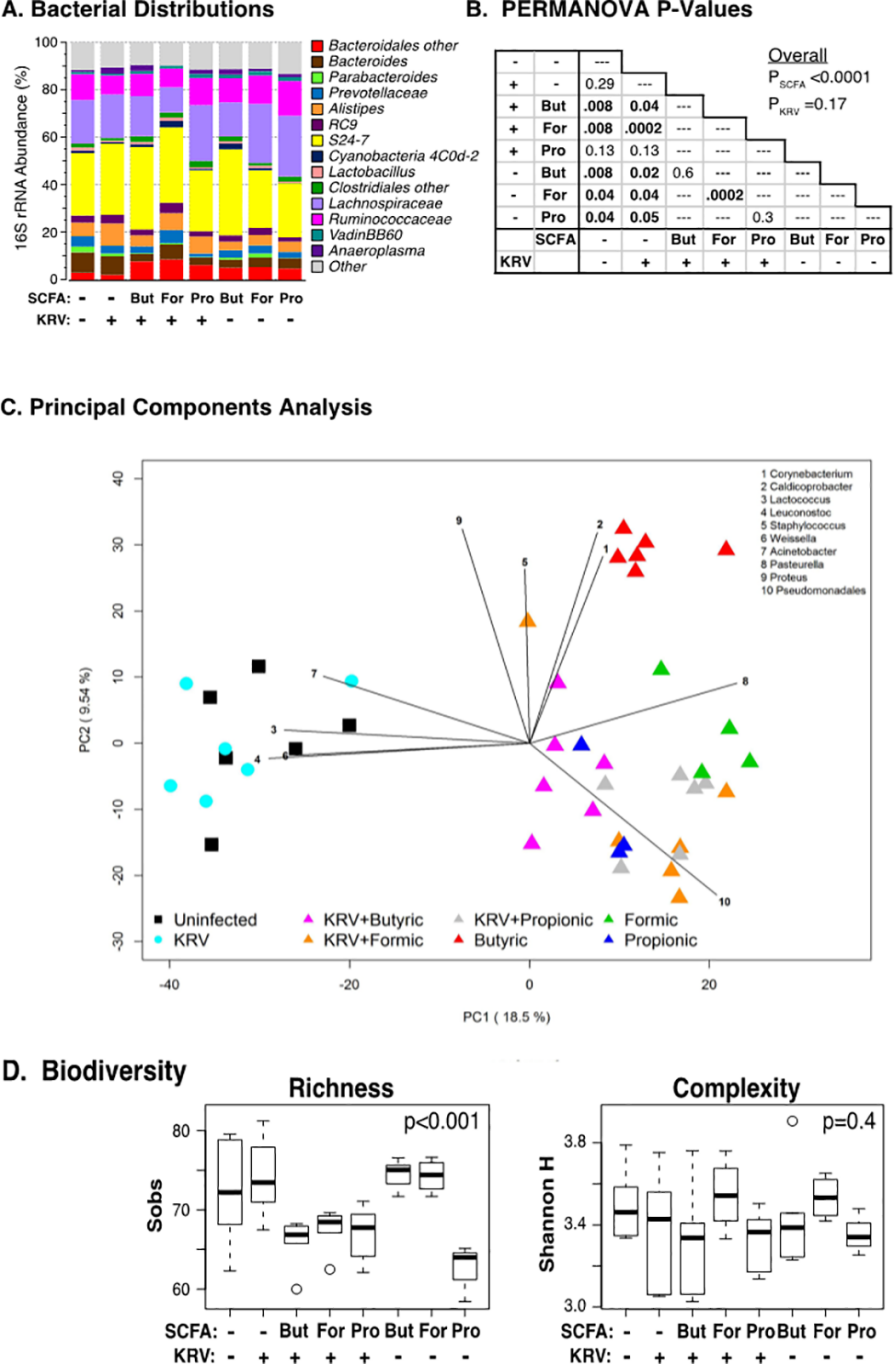
Bacterial taxa in animals treated with SCFAs. Rats were divided into eight groups and were left untreated (n = 6); administered KRV only (n = 6); or administered KRV plus 250 mM of butyrate (n = 6), formate (n = 6), or propionate (n = 6); or butyrate (n = 6), formate (n = 4), or propionate (n = 3) only. Fecal DNA was extracted from feces collected on day 5 post-infection and analyzed for bacterial composition. Panel A: Stacked bar chart of median percent counts of Operational Taxonomic Units (OTU) representing bacterial genera with a frequency of ≥1% of total counts in fecal samples from rats treated with SCFAs as indicated in the figure using Protocol 1 as described in Fig 1. Panel B: Results of PERMANOVA tests across all treatment groups (under the “Overall” heading) and pairs of treatment groups (table). Panel C: The PCA scores are displayed for each sample. The different colors and shapes correspond to the various treatment groups as indicated in the figure and described in *Materials and Methods*. Vectors corresponding to the indicated top 10 genera with the highest loadings are displayed. The magnitude and direction correspond to the weights. Panel D: The distribution of Sobs and Shannon diversity indices across groups. The area inside each box represents the interquartile range (IQR: 25th to 75th percentiles), the median is denoted by a line. The whiskers extend 1.5 IQR from the box, the observations outside of this range are displayed as points.

Because our previous work demonstrated that T1D progression in KRV-infected LEW1.WR1 rats is associated with an altered intestinal microbiome [5], and considering the effect that the maternal diet may have on the offspring gut microbiome [37–39], we analyzed the influence of SCFA treatment on the composition of the offspring intestinal microbiome. Rats were treated using Protocol 1 and infected with KRV at the age of 21–25 days (n = 3–6 per group). Fecal samples were collected on day 5 post-infection. Operational taxonomic units (OTUs) were produced by clustering sequences with identical taxonomic assignments. This process generated 15,034,637 high-quality 16S rRNA sequences for 43 samples (average sequence length: 297 nt; median sequence/sample: 339,580, range: 232,287 to 497,742). The median Goods coverage score was ≥ 99.994% at the rarefaction point of 237,113. We identified 157 individual genus-level taxa in the fecal samples. As expected (Fig 2A), the predominant bacterial taxa in the treated and untreated rats belonged to the phyla Bacteroidetes (genera *S24-7*, *Bacteroides*, and *Alistipes*) and Firmicutes (families *Lachnospiraceae* and *Ruminococcaceae*). However, SCFA treatment resulted in notable changes in the fecal microbiota, as evidenced by the following analyses.

PERMANOVA tests presented in Fig 2B revealed significant associations between genus-level microbiome composition and SCFA treatment (*p*<0.0001) but no or small effects of KRV infection when analyzed alone (*p* = 0.17) or as a covariate with SCFA treatment (*p* = 0.06). Furthermore, an exploratory principal component analysis (PCA) indicated that SCFA-treated and untreated animals clustered separately along the first PC axis and that no separation was evident between KRV-infected and uninfected animals (Fig 2C and S1 Table). Among the untreated animals, little separation was evident between KRV-infected and uninfected animals. Similar results were found in analyzing phylum- and family-level operational taxonomic unit (OTU) datasets. Biplot analysis shown in Fig 2C indicated that several taxa influenced the split between SCFA-treated and untreated animals, including *Lactococcus*, *Leuconostoc*, *Weissella*, and *Acinetobacter* (enriched in SCFA-untreated groups) as well as *Pasteurella* and *Pseudomonadales* (enriched in SCFA-treated groups).

Both PCA analysis and PERMANOVA tests of pairs of treatment groups revealed complex effects of particular SCFAs and of KRV infection on the microbiome structure (Fig 2B and Fig 2C). The group administered butyric acid only, for example, clustered with the other SCFA-treated groups with respect to PC1 but separately from the other SCFA-treated groups with respect to PC2, with higher levels of *Corynebacterium* than all other groups. The majority of the remainder of the rats clustered together and tended to have gut microbiota composed of greater levels of *Acinetobacter* and *Pseudomonadales*. Together, the data provide further evidence that SCFA-induced protection from islet autoimmunity may involve the induction of unique communities of gut bacteria that differ from the gut bacteria in rats that either did not receive any SCFA treatment or were administered KRV only.

Lastly, the Sobs (the number of species observed) biodiversity index, which measures the OTU counts in each sample, differed significantly (*p*<0.0001) across all treatment groups (Fig 2D). Not only did both KRV infection (*p* = 0.01) and SCFA species (*p* = 0.003) contribute as main effects but also the KRV x SCFA interaction term was also significantly (*p*<0.001) associated with Sobs. In contrast, the Shannon alpha diversity that takes in account the richness of a sample (The number of species) and the evenness of taxa in the sample (The relative prevalence of the various OTUs within the community) was comparable between groups (*p* = 0.4) and was not affected by KRV (*p* = 0.3) or SCFA treatment (*p* = 0.2). Together, the 16S sequence data provide evidence that SCFA therapy alters the overall composition and diversity of intestinal bacterial communities among treatment groups.

### SCFA treatment alters the abundance of individual bacterial taxa

We used Protocol 1 to investigate whether SCFA therapy alters the abundances of individual gut bacteria. The rats were divided into eight groups and were left untreated (n = 6); administered KRV only (n = 6); or administered KRV plus 250 mM of butyrate (n = 6), formate (n = 6), or propionate (n = 6); or butyrate (n = 6), formate (n = 4), or propionate (n = 3) only. Altogether, the data indicate that SCFA therapy induced significant alterations in the abundance of bacterial taxa belonging to the phyla Firmicutes (28 taxa), Proteobacteria (16 taxa), Bacteroidetes (6 taxa), and Actinobacteria (3 taxa) as of day 5 post-infection (Table 1 and S2 Table). Among the Firmicutes phylum, there was a substantial reduction in the median abundances of *Leuconostoc*, *Weissella*, *Lactococcus*, *Peptostreptococcaceae*, *Clostridium*, *and Turicibacter* in rats treated with KRV plus all three SCFAs versus KRV only. The data also demonstrate that the abundances of 14 Firmicutes taxa were altered in KRV versus the uninfected control group (Table 1). Among the Bacteroidetes phylum, all three SCFAs reduced the abundance of *Bacteroides* and increased the abundances of *Bacteroidales* and *Porphyromonadaceae*. The SCFA-induced alterations in the Proteobacteria phylum were linked with increased abundances of taxa such as *Escherichia-Shi*, *Enterobacteriaceae*, *Enterobacter*, and *Proteobacteria* and a decrease in *Acinetobacter* and *Haemophilus*. Finally, we observed that therapy with SCFAs reversed KRV-induced alterations in the abundance of a number of taxa, such as the Firmicutes genera *Leuconostoc*. *Weissella*, and *Lactococcus*. Collectively, these observations suggest that SCFA treatment substantially modulates the abundance of bacterial communities in KRV-infected and uninfected animals.

**Table 1:** Median abundance of bacterial communities in LEW1.WR1 rats treated with SCFAs.

Taxa	Uninfected	KRV	KRV plus Butyrate	KRV plus Formate	KRV plus Propionate	Butyrate	Formate	Propionate
	(N = 6)	(N = 6)	(N = 6)	(N = 6)	(N = 6)	(N = 6)	(N = 4)	(N = 3)
**Firmicutes**								
*Leuconostoc*	**0.003**	**0.004**^¶^	**0.000**	**0.000**	**0.000**	**0.000**	**0.000**	**0.000**
*Weissella*	**0.001**	**0.001**	**0.000**	**0.000**	**0.000**	**0.000**	**0.000**	**0.000**
*Lactococcus*	**0.002**	**0.003**	**0.000**	**0.000**	**0.000**	**0.000**	**0.000**	**0.000**
*Peptostreptococcaceae*	**1.369**	**1.094**	**0.620**	**0.216**	**0.537**	**1.284**	**0.255**	**0.436**
*Clostridium*	**0.011**	**0.017**	**0.005**	**0.000**	**0.003**	**0.006**	**0.000**	**0.001**
*Turicibacter*	**0.686**	**1.280**	**0.133**	**0.004**	**0.326**	**0.741**	**0.062**	**0.122**
*Coprococcus*	**0.028**	**0.096**	**0.048**	**0.055**	**0.027**	**0.055**	**0.072**	**0.063**
**Bacteroidetes**								
*Bacteroides*	**1.756**	**1.396**	**0.240**	**0.429**	**0.196**	**0.870**	**1.858**	**0.190**
*Bacteroidales*	**2.521**	**2.191**	**7.532**	**7.897**	**6.521**	**5.145**	**5.203**	**4.480**
*Porphyromonadaceae*	**0.030**	**0.004**	**0.662**	**1.284**	**1.171**	**0.315**	**0.378**	**0.579**
*Butyricimonas*	**0.123**	**0.423**	**0.284**	**0.748**	**0.264**	**0.623**	**0.564**	**0.225**
**Proteobacteria**								
*Acinetobacter*	**0.001**	**0.001**	**0.000**	**0.000**	**0.000**	**0.000**	**0.000**	**0.000**
*Haemophilus*	**0.006**	**0.008**	**0.002**	**0.011**	**0.004**	**0.047**	**0.029**	**0.029**
*Pasteurella*	**0.000**	**0.000**	**0.000**	**0.006**	**0.000**	**0.002**	**0.028**	**0.000**
*Pseudomonadales*	**0.000**	**0.000**	**0.000**	**0.022**	**0.003**	**0.000**	**0.007**	**0.004**
*Pseudomonas*	**0.000**	**0.000**	**0.002**	**0.002**	**0.000**	**0.000**	**0.001**	**0.000**
*Escherichia-Shi*	**0.017**	**0.031**	**0.083**	**0.504**	**0.078**	**0.121**	**0.300**	**0.193**
*Enterobacteriaceae*	**0.004**	**0.003**	**0.012**	**0.010**	**0.002**	**0.024**	**0.011**	**0.002**
*Enterobacter*	**0.003**	**0.004**	**0.010**	**0.069**	**0.012**	**0.020**	**0.045**	**0.008**
*Proteobacteria*	**0.004**	**0.001**	**0.010**	**0.009**	**0.007**	**0.006**	**0.014**	**0.003**

^¶^The highlights in the KRV column represent significant differences (adjusted FDR *p* values <0.1) compared with uninfected rats; the highlights in the KRV plus SCFAs columns represent significant differences versus KRV; the highlights in the SCFAs only columns represent significant differences compared with the uninfected control. Shown in brackets are the number of animals per group.

### SCFA treatment reverses virus-induced alterations in the gut microbiome

**Fig 3:**
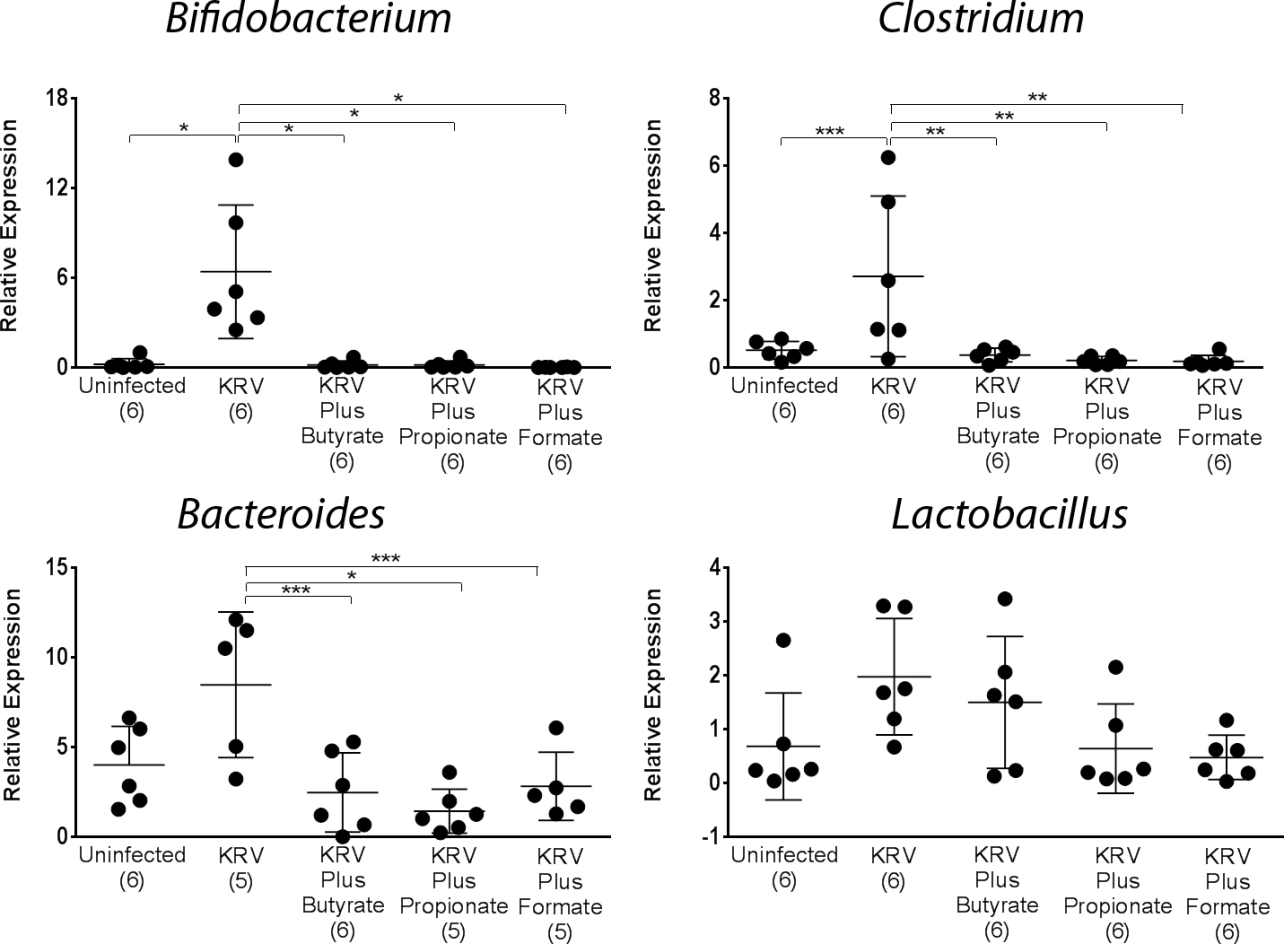
PCR analysis of bacterial abundances in the intestine from SCFA-treated animals. Rats were treated with KRV plus SCFAs using Protocol 1. DNA was extracted from fecal samples collected from individual rats on day 5. Quantitative PCR analysis was used to evaluate the level of various bacterial genera, as indicated in the figure. Standard curves were obtained using DNA extracted from a fecal sample from a B6 mouse. Shown are relative DNA levels that were calculated using a bacterial reference gene. Dots represent individual rats, and the lines represent the mean and SD. The numbers in brackets indicate the animal number. Statistical analyses were performed using ANOVA with Bonferroni's multiple comparison adjustments. *p < 0.001; **p < 0.01; ***p < 0.05.

Given the high-throughput observations that SCFAs exert on the gut microbiota of LEW1.WR1 rats, we used quantitative PCR analysis to validate our high-throughput observations. We were particularly interested in defining whether SCFAs could reverse virus-induced alterations in the gut microbiome. For this purpose, we used quantitative PCR analysis to evaluate the abundance of bacterial genera that we previously found to be modulated by KRV [5] or by SCFA (Table 1) on day 5 post-infection (*n* = 3–6 per group). The data presented in Fig 3 demonstrate that as we previously reported [5], infection with KRV increased the level of *Bifidobacterium* and *Clostridium* but not that of *Bacteroides* and *Lactobacillus* compared with untreated control animals (*p* < 0.001 and *p* < 0.01, respectively). Moreover, in results similar to the high throughput data, therapy commencing prenatally resulted in a complete reduction in the abundance of *Clostridium* and *Bifidobacterium* versus animals administered KRV only (*p* < 0.001 for both). These findings suggest that SCFA therapy can override virus-induced alterations in the gut microbiota.

### SCFA therapy down-modulates KRV-induced inflammation in the intestine

**Fig 4:**
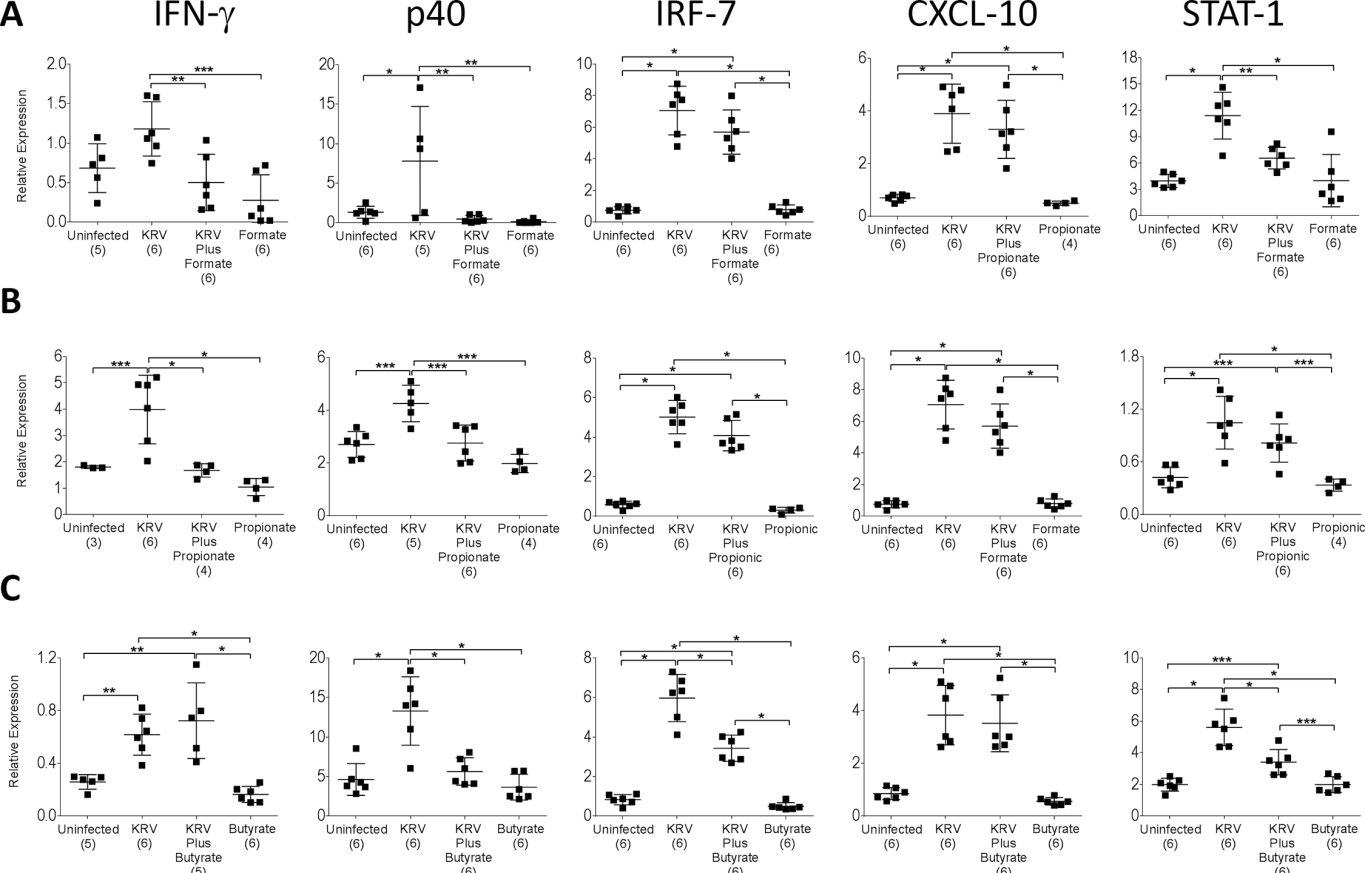
Gene expression in the Peyer’s patches from SCFA-treated LEW1.WR1 rats. LEW1.WR1 rats were treated with KRV plus formate (A), Propionate (B), or Butyrate (C) using Protocol 1. RNA was extracted from Peyer’s patches 5 days after virus infection and the expression levels of the indicated genes were assessed using quantitative RT-PCR. The results are expressed as the mRNA expression of the gene of interest relative to the expression of β-actin. Shown are data from individual rats. The lines represent the mean and SD. The numbers in brackets indicate the animal number. Statistical analyses were performed using an ANOVA with Bonferroni's multiple comparison adjustments. *p < 0.001; **p < 0.01; ***p < 0.05.

We recently postulated that virus-induced innate immune activation in the intestine may play a role in the induction of islet autoimmunity in the LEW1.WR1 rat model [5]. We tested the hypothesis that SCFAs protect LEW1.WR1 rats from islet autoimmunity via a mechanism that could be associated with the down-modulation of virus-induced proinflammatory responses in Peyer’s patches. For this purpose, we measured the transcript levels of IFN-γ, the p40 subunit of IL-12 and IL-23, IRF-7, CXCL-10, and STAT-1 in rats treated with KRV plus SCFAs using Protocol 1. Peyer’s patches from animals that did not receive any treatments or were treated with KRV only were used as a control (*n* = 5–6 per group). The data presented in Fig 4 demonstrate that, consistently with our previous data [5], infection with KRV increased the transcript levels of all the above genes compared with the uninfected controls, whereas treatment with KRV plus formate or propionate reduced the expression of IFN-γ, p40, or STAT-1 compared with KRV only. Diminished levels of transcripts for p40, IRF-7, and STAT-1 were also detected in Peyer’s patches from animals administered KRV plus butyrate. These data imply that SCFA-induced disease prevention could be associated with downregulation of virus-induced inflammation in the intestine.

### SCFA therapy alters lymphocyte compartments in Peyer’s patches

**Fig 5:**
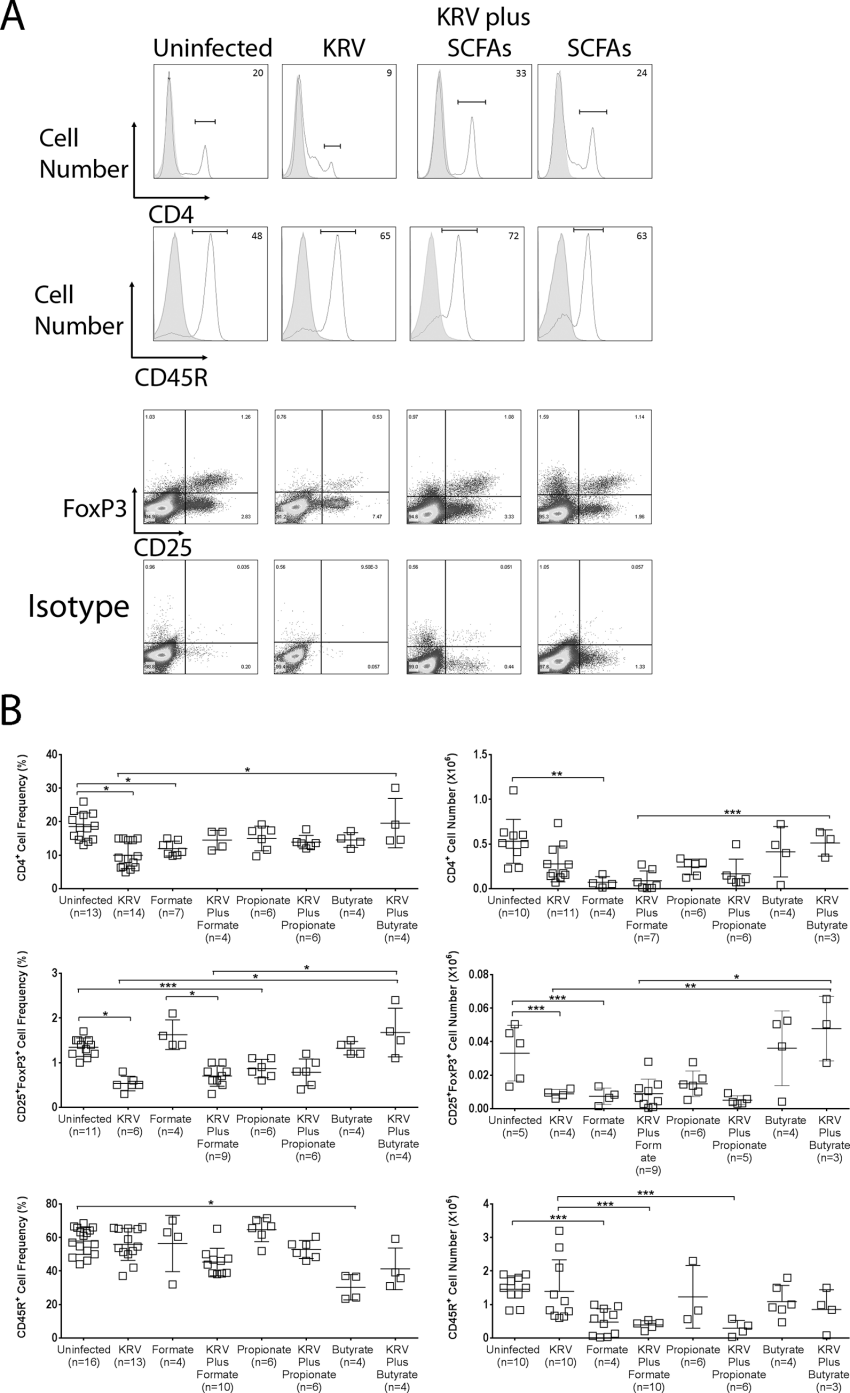
T and B cell frequencies and numbers in the Peyer’s patches from LEW1.WR1 rats treated with SCFAs. Rats were treated with SCFAs and were infected with KRV using Protocol 1. On day 5, cells from the Peyer’s patches were harvested, counted, and stained with fluorochrome-conjugated mAbs directed against CD4, CD25, Foxp3, and CD45R, followed by flow cytometry analysis. Panel A includes representative histograms of lymphocyte subsets in the Peyer’s patches from rats treated with sodium butyrate. The shaded areas show staining with the isotype control. The frequency of cells stained is indicated in the upper right corner. Data shown in Panel B represent the frequency and cell number of the indicated subsets found in Peyer’s patches from individual rats. The lines in Panel B represent the mean and SD. The numbers in brackets indicate the animal number. Statistical analyses were performed using an ANOVA with Bonferroni's multiple comparison adjustments. *p < 0.001; **p < 0.01; ***p < 0.05.

It was recently reported that SCFA therapy provides protection against colitis via the upregulation of Treg cells [40, 41]. Moreover, we hypothesized that antibiotic-induced disease prevention in the LEW1.WR1 rat may be linked with altered adaptive immunity in Peyer’s patches [5]. Thus, we tested the possibility that SCFA-induced disease prevention in LEW1.WR1 rats is potentially linked with modulation of the T and/or B cell compartments in Peyer’s patches. To do so, animals were treated with SCFAs using Protocol 1 (*n* = 3–16 per group). The control groups included rats treated with KRV only or SCFAs only. Peyer’s patches were harvested 5 days after virus inoculation and analyzed by flow cytometry for the expression of CD4, CD8, and CD45R (a marker of B lymphocytes in the rat) and the co-expression of CD25 and Foxp3 (Treg cells). Fig 5 indicates that infection with KRV led to a 2-fold reduction in the frequency of CD4^+^ cells compared with the uninfected control (*p* < 0.001). Treatments with formate and propionate did not modulate the percentage of CD4^+^ cells, whereas therapy with butyrate resulted in a 2-fold increase in the percentage of CD4^+^ cells compared with KRV only (*p* < 0.05). The CD4^+^ cell number was reduced in the Peyer’s patches of animals treated with KRV only compared with naïve rats (*p* < 0.001). We further noted that the frequency of Treg cells was reduced by 60% in rats treated with KRV only compared with uninfected animals (*p* < 0.001). The proportion of Treg cells in rats treated with KRV plus butyrate but not formate or propionate was significantly increased compared to rats administered KRV only (*p* < 0.001). A 75% reduction in the total Treg cell number was detected in rats injected with KRV versus the uninfected control (*p* < 0.05). Treatment with KRV plus butyrate but not formate or propionate led to a 5-fold increase in Treg cell numbers compared to KRV only (*p* < 0.001). Finally, the number of B cells was significantly reduced in the Peyer’s patches of animals administered KRV plus formate or propionate but not KRV plus butyrate compared to KRV only (*p* < 0.05 for both). Taken together, these findings could imply that SCFA therapy alters the frequency and/or numbers of T and B cells in Peyer’s patches.

## Discussion

Growing evidence indicates that lifestyle changes in the last 50 years, particularly the ‘Western’ diets, which are high in protein and fat but low in fiber have altered the genetic composition and metabolic activity of the gut microbiota presumably leading to a dramatic rise in metabolic and immune-mediated disorders [15]. SCFAs that are the end product of the bacterial fermentation of indigestible dietary components such as plant fiber [18] play a key role in maintaining gut health (reviewed in ref. [42]). Because SCFAs enter the systemic circulation, they may influence metabolic and immune pathways via their effects on various cell types [43, 44]. Herein, we tested the effect of SCFA therapy on the gut microbiota, virus-induced intestinal immunity and disease progression. Our studies demonstrate several key findings that underscore the therapeutic potential of SCFAs in diabetes. First, SCFAs ameliorate virus-induced islet autoimmunity in the offspring if provided to rat mothers prior to pregnancy but not after weaning. Second, SCFAs reshape the intestinal microbiome and override virus-induced alterations in the abundance of a number of bacterial taxa. Third, SCFA therapy modulates innate and adaptive immunity in the intestine without compromising the ability of the host immune system to eradicate the virus (this manuscript and S1 Fig).

Our studies demonstrate that treating rat breeders with SCFAs leads to profound alterations in the gut microbiota of the offspring. This is consistent with human data showing that dietary intake, especially of non-digestible carbohydrates, can modulate the intestinal microbiome. For example, the gut microbiota from children from rural villages in Africa, who consumed high amounts of plant fiber, had low abundances of Firmicutes and elevated levels of Bacteroidetes compared with children from Europe (reviewed in ref. [45]). Our PCR and high-throughput sequencing data further indicate that SCFA treatment results in reduced abundances of *Clostridium* and *Bifidobacterium* compared with animals administered with virus only. The reduction in these taxa in the SCFA-treated animals is reminiscent of our earlier studies demonstrating a correlation between a reduction in the abundances of these genera and diabetes prevention following therapy with blockers of innate immunity [46, 47] and antibiotics [5].

Due to the tight interdependence between the gut microbiota and the immune system [48, 49], it is plausible to hypothesize that the changes detected in the gut bacterial composition in animals treated with SCFAs could be secondary to the effect of these metabolites on the immune system. It may also be that these alterations are linked with changes in the abundances of other yet unidentified bacterial groups. Finally, it may be hypothesized that the SCFA treatment regimen we have used favors the growth of particular taxa or perhaps exerts a toxic effect on certain bacteria, as has been previously suggested [50].

Our data indicate that administration of SCFAs alters virus-induced innate immunity. We observed that SCFAs downregulate KRV-induced proinflammatory cytokine and chemokine gene expression in Peyer’s patches. These observations raise the hypothesis that downregulation of inflammation in the intestine may be linked with the mechanism by which SCFAs restore immune tolerance. This is consistent with our earlier data demonstrating that downmodulation of virus-induced innate immunity with anti-inflammatory agents such as steroids [51], histone deacetylase blockers [47], or antibiotics [5] exerts a beneficial effect on diabetes. Our findings are also consistent with previous reports that SCFAs modulate the expression of cytokines, chemokines, and adhesion molecules in neutrophils and endothelial cells, an effect that could be linked with the ability of SCFAs to alter leukocyte recruitment to sites of inflammation [42]. Lastly, it is unclear why in contrast to formate and propionate, therapy with butyrate failed to reduce the level of IFN-γ in the intestine. It could be that formate and propionate are more efficient in suppressing IFN-γ signaling pathways compared with butyric acid.

We further observed that SCFA administration modulates the B or Treg cell compartments in Peyer’s patches. B lymphocytes could play an important role in virus-induced inflammation, as they are activated by KRV to produce high amounts of proinflammatory cytokines [24]. Treg cells were recently shown to be linked with the mechanism of butyrate-induced prevention of inflammatory bowel disease in mice [40, 41]. Our observation that butyrate, but not formate or propionate, induces Treg cells is consistent with a recently published report demonstrating that butyric acid is highly efficient in inducing Tregs [40].

A number of questions remain unresolved. First, how therapy with SCFAs that commences prenatally but not after weaning protects from T1D is unclear. Second, we have not yet identified the critical time window for SCFA-induced diabetes amelioration. It may be that prevention of islet autoimmunity is associated at least in part with the substantial alterations induced by SCFAs in gut bacteria via breast feeding or fecal transfer from mothers to offspring via coprophagy [37–39]. Our PCR data from rats administered with SCFAs beginning at weaning allude to the possibility that SCFAs may exert different effects on the gut microbiota when provided early versus late in life.

Previous studies that examined the ability of diets that promote SCFA production to modulate diabetes in the NOD mouse model led to mixed data. NOD mice fed with the fermentable plant fibers pectin and xylan were shown to have elevated levels of SCFAs in the colon but increased diabetes incidence [52]. Our data are compatible with observations from another study demonstrating that feeding NOD mice diets, which promote the production of large amounts of acetate or butyrate prevent diabetes via mechanisms associated with modulation of adaptive immunity [53].

In conclusion, our data imply that therapy with SCFAs substantially alters the intestinal microbiome and protects LEW1.WR1 rats from virus-induced islet destruction, thus representing an attractive therapeutic approach to diabetes prevention. Future studies in genetically-susceptible individuals will be required to determine whether SCFAs can prevent the human disease.

## Supporting information

S1 Table The contribution of taxa to the components of the PCA. (DOCX)

S2 Table Results from Wilcoxon tests indicating the pairwise comparisons for taxa with a statistically significant difference across groups (overall p-value). (DOCX)

S1 Fig KRV-specific adaptive immunity and virus clearance in rats treated with SCFAs. Rats were either left untreated, were injected with KRV, or were administered with KRV plus SCFA using treatment protocol 1. Panels A and B: Spleen cells were harvested on day 12 following infection and activated in the presence of KRV (*n* = 3–9 per group). Representative flow cytometry images and frequencies of IFN-γ^+^ cells out of the total CD8^+^ cells are shown in Panels A and B, respectively. The horizontal (IFN-γ) and vertical (CD8) axes indicate the fluorescence intensity. The proportion of CD8^+^ cells out of the total IFN-γ^+^ cells is shown in the upper right quadrant of each flow panel. Panels C: Blood samples were removed on days 12 and 40 (*n* = 2–3 per group). The serum was diluted as indicated in the figure and was assayed for the presence of virus-specific Abs. Each bar represents the mean value. Panel D: Blood, spleens, and pancreatic LNs were removed 5 and 40 days after infection ((*n* = 5–6 per group). RNA was extracted, and the level of KRV transcripts was determined by quantitative RT-PCR. The results are expressed as the expression of the gene mRNA relative to the expression of β-actin. Statistical analyses were performed using an ANOVA with Bonferroni's multiple comparison adjustments. *p < 0.001; **p < 0.01; ***p < 0.05.
(DOCX)
