# Supplementary material for: Maternal treatment with short-chain fatty acids modulates the intestinal microbiota and immunity and ameliorates type 1 diabetes in the offspring
Source: PLoS One. 2017 Sep 8;12(9):e0183786. doi: 10.1371/journal.pone.0183786 (PMC5590848; doi:10.1371/journal.pone.0183786)
Supplement: S1 Table — (DOCX) [file pone.0183786.s001.docx]

| Supporting Information **S1 Table.** The contribution of taxa to the components of the PCA | | | | |
| --- | --- | --- | --- | --- |
| **Taxa** | **PC1** | **PC2** |  |  |
| Actinomyces | -0.03735 | -0.02273 |  |  |
| Arthrobacter | -0.02383 | -0.01042 |  |  |
| Bifidobacterium | -0.08835 | 0.093525 |  |  |
| Brevibacterium | 0.026621 | -0.00913 |  |  |
| Collinsella | -0.01097 | -0.0327 |  |  |
| Coriobacteriaceae | -0.01726 | -0.02256 |  |  |
| Corynebacterium | 0.085908 | 0.297599 |  |  |
| Enterorhabdus | -0.00514 | -0.01198 |  |  |
| Janibacter | 0.031347 | -0.03377 |  |  |
| Micrococcales | -0.00856 | -0.01456 |  |  |
| Propionibacteriu | -0.10896 | -0.05614 |  |  |
| Rhodococcus | 0.002556 | -0.02039 |  |  |
| Rothia | -0.0338 | 0.152102 |  |  |
| Slackia | -0.05287 | -0.04034 |  |  |
| Alistipes | -0.00103 | -0.03524 |  |  |
| Bacteroidales | 0.023588 | -0.02756 |  |  |
| Bacteroides | -0.01054 | -0.0295 |  |  |
| Bacteroidetes | 0.000363 | -0.03005 |  |  |
| Barnesiella | 0.043507 | -0.00407 |  |  |
| Butyricimonas | 0.018619 | -0.01221 |  |  |
| Chryseobacterium | -0.07799 | -0.00679 |  |  |
| Cloacibacterium | -0.03609 | -0.0132 |  |  |
| Flavobacteriaceae | -0.05043 | 0.001732 |  |  |
| Flavobacterium | -0.01011 | -0.01187 |  |  |
| Odoribacter | 0.059599 | -0.06046 |  |  |
| Parabacteroides | -0.01713 | 0.010173 |  |  |
| Paraprevotella | 0.19178 | -0.03003 |  |  |
| Porphyromonadaceae | 0.081014 | -0.04751 |  |  |
| Prevotella | -0.00695 | -0.02522 |  |  |
| Prevotellaceae | 0.000168 | -0.02523 |  |  |
| RC9-gut-group | 0.00069 | -0.02638 |  |  |
| Rikenella | 0.00676 | -0.03895 |  |  |
| Rikenellaceae | -0.04306 | -0.06137 |  |  |
| S24-7 | 0.003981 | -0.01758 |  |  |
| Sphingobacteriaceae | -0.01011 | -0.01187 |  |  |
| ratAN060301C | 0.013208 | 0.013927 |  |  |
| Candidate-division-TM7 | 0.065129 | -0.00422 |  |  |
| 4C0d-2 | 0.031728 | -0.00561 |  |  |
| Chloroplast | -0.07419 | -0.07758 |  |  |
| Mucispirillum | 0.046692 | -0.01432 |  |  |
| Fibrobacter | 0.018621 | -0.01213 |  |  |
| Acetitomaculum | 0.157151 | -0.1167 |  |  |
| Aerococcus | 0.09366 | 0.239849 |  |  |
| Allobaculum | -0.08919 | -0.0877 |  |  |
| Anaerococcus | -0.00695 | -0.02522 |  |  |
| Anaerofilum | 0.051226 | -0.00036 |  |  |
| Anaerofustis | -0.02379 | -0.21816 |  |  |
| Anaerosporobacter | -0.02646 | 0.002473 |  |  |
| Anaerostipes | 0.137947 | 0.239781 |  |  |
| Anaerotruncus | 0.003452 | -0.03605 |  |  |
| Anaerovorax | 0.117354 | 0.008129 |  |  |
| Bacilli | 0.070335 | 0.193791 |  |  |
| Bacillus | 0.033688 | 0.033405 |  |  |
| Blautia | -0.18739 | 0.064661 |  |  |
| Butyrivibrio | 0.022571 | -0.07375 |  |  |
| Caldicoprobacter | 0.07926 | 0.335152 |  |  |
| Candidatus-Arthromitus | 0.129802 | -0.09392 |  |  |
| Carnobacteriaceae | -0.00188 | -0.02519 |  |  |
| Christensenella | -0.00631 | 0.010108 |  |  |
| Christensenellaceae | -0.00032 | 0.006476 |  |  |
| Clostridiaceae | -0.03307 | 0.021666 |  |  |
| Clostridiales | 0.014675 | -0.03179 |  |  |
| Clostridium | -0.05344 | 0.16586 |  |  |
| Coprococcus | -0.00356 | -0.02044 |  |  |
| Dialister | -0.01097 | -0.0327 |  |  |
| Dorea | -0.00739 | -0.02709 |  |  |
| Enterococcus | -0.0057 | 0.061768 |  |  |
| Erysipelotrichaceae | -9.30E-06 | -0.02276 |  |  |
| Faecalibacterium | 0.138049 | 0.001569 |  |  |
| Family-XIII-Incertae-Sedis | 0.000828 | -0.01569 |  |  |
| Firmicutes | 0.002763 | -0.00317 |  |  |
| Flavonifractor | -0.01423 | -0.05502 |  |  |
| Jeotgalicoccus | 0.078284 | 0.206281 |  |  |
| Lachnospira | 0.00861 | -0.01421 |  |  |
| Lachnospiraceae | 0.005936 | -0.02647 |  |  |
| Lactobacillales | 0.012114 | -0.08064 |  |  |
| Lactobacillus | 0.005508 | -0.0183 |  |  |
| Lactococcus | -0.28697 | 0.021176 |  |  |
| Leuconostoc | -0.30516 | -0.02423 |  |  |
| Marvinbryantia | -0.03188 | 0.087543 |  |  |
| Megamonas | -0.00695 | -0.02522 |  |  |
| Oribacterium | 0.002747 | -0.01141 |  |  |
| Oscillibacter | 0.018901 | -0.03317 |  |  |
| Papillibacter | 0.000355 | 0.126023 |  |  |
| Peptococcaceae | -0.00796 | -0.03048 |  |  |
| Peptococcus | 0.004086 | -0.01705 |  |  |
| Peptostreptococcaceae | -0.01427 | 0.000936 |  |  |
| Pseudobutyrivibrio | 0.05673 | -0.09982 |  |  |
| Roseburia | -0.01315 | -0.03857 |  |  |
| Ruminococcaceae | 0.008699 | -0.02554 |  |  |
| Ruminococcus | -0.00866 | -0.0039 |  |  |
| Shuttleworthia | 0.015461 | -0.02066 |  |  |
| Staphylococcus | -0.00529 | 0.277096 |  |  |
| Streptococcaceae | -0.14042 | 0.15546 |  |  |
| Streptococcus | 0.008494 | 0.012946 |  |  |
| Subdoligranulum | -0.11546 | 0.018586 |  |  |
| Thermicanus | 0.023272 | -0.04367 |  |  |
| Turicibacter | -0.05639 | 0.044361 |  |  |
| Veillonella | -0.03602 | -0.02323 |  |  |
| Weissella | -0.27374 | -0.01888 |  |  |
| vadinBB60 | 0.022062 | -0.0326 |  |  |
| Fusobacterium | 0.008706 | 0.046709 |  |  |
| Acidovorax | -0.05861 | -0.0036 |  |  |
| Acinetobacter | -0.24134 | 0.106757 |  |  |
| Aeromonas | -0.01237 | -0.01084 |  |  |
| Alcaligenes | -0.01011 | -0.01187 |  |  |
| Alphaproteobacteria | -0.05164 | -0.0254 |  |  |
| Arcobacter | -0.05975 | -0.05675 |  |  |
| Betaproteobacteria | -0.15552 | -0.02945 |  |  |
| Bilophila | 0.045961 | -0.07027 |  |  |
| Brevundimonas | -0.02472 | -0.02008 |  |  |
| Burkholderiales | -0.00695 | -0.02522 |  |  |
| Citrobacter | -0.18935 | -0.08278 |  |  |
| Comamonadaceae | -0.10593 | -0.00577 |  |  |
| Comamonas | -0.11128 | 0.005139 |  |  |
| Cupriavidus | 0.002747 | -0.01141 |  |  |
| Delftia | -0.08881 | -0.00829 |  |  |
| Desulfovibrio | 0.018735 | -0.02069 |  |  |
| Desulfovibrionaceae | 0.025249 | 0.015895 |  |  |
| Enhydrobacter | -0.11509 | -0.03609 |  |  |
| Enterobacter | 0.074573 | -0.01468 |  |  |
| Enterobacteriaceae | 0.053134 | 0.019837 |  |  |
| Escherichia-Shi | 0.054154 | -0.02142 |  |  |
| Gammaproteobacteria | 0.121351 | -0.07147 |  |  |
| Gemmobacter | 0.114424 | 0.076383 |  |  |
| Haemophilus | 0.019761 | 0.002656 |  |  |
| Hydrogenophilus | 0.028003 | -0.04515 |  |  |
| Kluyvera | -0.03346 | -0.03554 |  |  |
| Kordiimonas | 0.037986 | -0.00569 |  |  |
| Legionella | -0.00546 | -0.0295 |  |  |
| Moraxella | 0.031582 | 0.015099 |  |  |
| Morganella | -0.00188 | -0.02519 |  |  |
| Neisseriaceae | -0.01011 | -0.01187 |  |  |
| Novosphingobium | -0.00632 | -0.03566 |  |  |
| Ochrobactrum | -0.00188 | -0.02519 |  |  |
| Pantoea | 0.027995 | -0.03052 |  |  |
| Paracoccus | -0.01097 | -0.0327 |  |  |
| Parasutterella | -0.02213 | -0.00541 |  |  |
| Pasteurella | 0.242503 | 0.095279 |  |  |
| Pasteurellaceae | 0.060371 | -0.00788 |  |  |
| Pectobacterium | -0.03719 | -0.0548 |  |  |
| Polaromonas | 0.002556 | -0.02039 |  |  |
| Proteobacteria | 0.094542 | 0.016869 |  |  |
| Proteus | -0.07886 | 0.340815 |  |  |
| Pseudomonadales | 0.219054 | -0.24189 |  |  |
| Pseudomonas | 0.210581 | -0.00243 |  |  |
| Ralstonia | 0.027998 | -0.04655 |  |  |
| Rhizobium | 0.057837 | -0.06638 |  |  |
| Stenotrophomonas | 0.110652 | -0.01969 |  |  |
| Thalassospira | 0.032189 | 0.014644 |  |  |
| Undibacterium | 0.022423 | -0.0326 |  |  |
| Xanthomonadaceae | -0.01011 | -0.01187 |  |  |
| Xanthomonas | 0.002747 | -0.01141 |  |  |
| mitochondria | 0.021705 | 0.020034 |  |  |
| Anaeroplasma | 0.000574 | 0.004185 |  |  |
| RF9 | 0.004458 | -0.0119 |  |  |
| Akkermansia | 0.097063 | 0.029239 |  |  |
|  |  |  |  |  |
