# Supplementary material for: Maternal treatment with short-chain fatty acids modulates the intestinal microbiota and immunity and ameliorates type 1 diabetes in the offspring
Source: PLoS One. 2017 Sep 8;12(9):e0183786. doi: 10.1371/journal.pone.0183786 (PMC5590848; doi:10.1371/journal.pone.0183786)
Supplement: S2 Table — (DOCX) [file pone.0183786.s002.docx]

**S2 Table.** Results from Wilcoxon tests indicating the pairwise comparisons for taxa with a statistically significant difference across groups (overall p-value)

| Taxon | Overall p-value | Comparison | p-value | FDR p-value* |
| --- | --- | --- | --- | --- |
| Leuconostoc | <.001 | Uninfected vs KRV | <.001 | <.001 |
|  |  | KRV vs KRV+Butyric | <.001 | <.001 |
|  |  | KRV vs KRV+Formic | <.001 | <.001 |
|  |  | KRV vs KRV+Propionic | <.001 | <.001 |
|  |  | KRV vs Butyric | <.001 | <.001 |
|  |  | KRV vs Formic | <.001 | <.001 |
|  |  | KRV vs Propionic | <.001 | <.001 |
|  |  | Uninfected vs KRV+Butyric | 1 | 1 |
|  |  | Uninfected vs Butyric | 1 | 1 |
|  |  | KRV+Butyric vs Butyric | 1 | 1 |
|  |  | Uninfected vs KRV+Formic | 1 | 1 |
|  |  | Uninfected vs Formic | 1 | 1 |
|  |  | KRV+Formic vs Formic | 1 | 1 |
|  |  | Uninfected vs KRV+Propionic | 1 | 1 |
|  |  | Uninfected vs Propionic | 1 | 1 |
|  |  | KRV+Propionic vs Propionic | 1 | 1 |
| Weissella | <.001 | Uninfected vs KRV | <.001 | <.001 |
|  |  | KRV vs KRV+Butyric | <.001 | <.001 |
|  |  | KRV vs KRV+Formic | <.001 | <.001 |
|  |  | KRV vs KRV+Propionic | <.001 | <.001 |
|  |  | KRV vs Butyric | <.001 | <.001 |
|  |  | KRV vs Formic | <.001 | <.001 |
|  |  | KRV vs Propionic | <.001 | <.001 |
|  |  | Uninfected vs KRV+Butyric | 1 | 1 |
|  |  | Uninfected vs Butyric | 1 | 1 |
|  |  | KRV+Butyric vs Butyric | 1 | 1 |
|  |  | Uninfected vs KRV+Formic | 1 | 1 |
|  |  | Uninfected vs Formic | 1 | 1 |
|  |  | KRV+Formic vs Formic | 1 | 1 |
|  |  | Uninfected vs KRV+Propionic | 1 | 1 |
|  |  | Uninfected vs Propionic | 1 | 1 |
|  |  | KRV+Propionic vs Propionic | 1 | 1 |
| Lactococcus | <.001 | KRV vs KRV+Butyric | <.001 | <.001 |
|  |  | KRV vs KRV+Formic | <.001 | <.001 |
|  |  | KRV vs KRV+Propionic | <.001 | <.001 |
|  |  | Uninfected vs KRV | <.001 | <.001 |
|  |  | KRV vs Butyric | <.001 | <.001 |
|  |  | KRV vs Formic | <.001 | <.001 |
|  |  | KRV vs Propionic | <.001 | <.001 |
|  |  | Uninfected vs KRV+Butyric | 0.206 | 0.332 |
|  |  | Uninfected vs KRV+Formic | 0.206 | 0.332 |
|  |  | Uninfected vs KRV+Propionic | 0.206 | 0.332 |
|  |  | Uninfected vs Butyric | 0.248 | 0.386 |
|  |  | Uninfected vs Formic | 0.248 | 0.386 |
|  |  | Uninfected vs Propionic | 0.248 | 0.386 |
|  |  | KRV+Butyric vs Butyric | 1 | 1 |
|  |  | KRV+Formic vs Formic | 1 | 1 |
|  |  | KRV+Propionic vs Propionic | 1 | 1 |
| Pseudomonadales | <.001 | Uninfected vs KRV+Butyric | <.001 | <.001 |
|  |  | Uninfected vs KRV+Formic | <.001 | <.001 |
|  |  | Uninfected vs KRV+Propionic | <.001 | <.001 |
|  |  | KRV vs KRV+Butyric | <.001 | <.001 |
|  |  | KRV vs KRV+Formic | <.001 | <.001 |
|  |  | KRV vs KRV+Propionic | <.001 | <.001 |
|  |  | KRV+Butyric vs Butyric | <.001 | <.001 |
|  |  | KRV+Formic vs Formic | <.001 | <.001 |
|  |  | KRV+Propionic vs Propionic | <.001 | <.001 |
|  |  | Uninfected vs KRV | 0.409 | 0.564 |
|  |  | KRV vs Butyric | 0.5 | 0.661 |
|  |  | KRV vs Formic | 0.5 | 0.661 |
|  |  | KRV vs Propionic | 0.5 | 0.661 |
|  |  | Uninfected vs Butyric | 1 | 1 |
|  |  | Uninfected vs Formic | 1 | 1 |
|  |  | Uninfected vs Propionic | 1 | 1 |
| Pasteurella | <.001 | KRV vs KRV+Butyric | <.001 | <.001 |
|  |  | KRV vs KRV+Formic | <.001 | <.001 |
|  |  | KRV vs KRV+Propionic | <.001 | <.001 |
|  |  | Uninfected vs KRV | <.001 | <.001 |
|  |  | KRV+Butyric vs Butyric | <.001 | <.001 |
|  |  | KRV+Formic vs Formic | <.001 | <.001 |
|  |  | KRV+Propionic vs Propionic | <.001 | <.001 |
|  |  | Uninfected vs Butyric | 0.002 | 0.012 |
|  |  | Uninfected vs Formic | 0.002 | 0.012 |
|  |  | Uninfected vs Propionic | 0.002 | 0.012 |
|  |  | Uninfected vs KRV+Butyric | 0.01 | 0.036 |
|  |  | Uninfected vs KRV+Formic | 0.01 | 0.036 |
|  |  | Uninfected vs KRV+Propionic | 0.01 | 0.036 |
|  |  | KRV vs Butyric | 0.24 | 0.376 |
|  |  | KRV vs Formic | 0.24 | 0.376 |
|  |  | KRV vs Propionic | 0.24 | 0.376 |
| Aerococcus | <.001 | Uninfected vs KRV | <.001 | <.001 |
|  |  | Uninfected vs KRV+Butyric | <.001 | <.001 |
|  |  | Uninfected vs KRV+Formic | <.001 | <.001 |
|  |  | Uninfected vs KRV+Propionic | <.001 | <.001 |
|  |  | Uninfected vs Butyric | <.001 | <.001 |
|  |  | Uninfected vs Formic | <.001 | <.001 |
|  |  | Uninfected vs Propionic | <.001 | <.001 |
|  |  | KRV vs KRV+Butyric | 1 | 1 |
|  |  | KRV vs Butyric | 1 | 1 |
|  |  | KRV+Butyric vs Butyric | 1 | 1 |
|  |  | KRV vs KRV+Formic | 1 | 1 |
|  |  | KRV vs Formic | 1 | 1 |
|  |  | KRV+Formic vs Formic | 1 | 1 |
|  |  | KRV vs KRV+Propionic | 1 | 1 |
|  |  | KRV vs Propionic | 1 | 1 |
|  |  | KRV+Propionic vs Propionic | 1 | 1 |
| Acinetobacter | <.001 | KRV vs KRV+Butyric | <.001 | <.001 |
|  |  | KRV vs KRV+Formic | <.001 | <.001 |
|  |  | KRV vs KRV+Propionic | <.001 | <.001 |
|  |  | KRV vs Butyric | <.001 | <.001 |
|  |  | KRV vs Formic | <.001 | <.001 |
|  |  | KRV vs Propionic | <.001 | <.001 |
|  |  | Uninfected vs KRV | <.001 | 0.002 |
|  |  | Uninfected vs KRV+Butyric | 0.019 | 0.055 |
|  |  | Uninfected vs KRV+Formic | 0.019 | 0.055 |
|  |  | Uninfected vs KRV+Propionic | 0.019 | 0.055 |
|  |  | Uninfected vs Butyric | 0.031 | 0.08 |
|  |  | Uninfected vs Formic | 0.031 | 0.08 |
|  |  | Uninfected vs Propionic | 0.031 | 0.08 |
|  |  | KRV+Butyric vs Butyric | 1 | 1 |
|  |  | KRV+Formic vs Formic | 1 | 1 |
|  |  | KRV+Propionic vs Propionic | 1 | 1 |
| Jeotgalicoccus | <.001 | Uninfected vs KRV | <.001 | <.001 |
|  |  | Uninfected vs Butyric | <.001 | <.001 |
|  |  | Uninfected vs Formic | <.001 | <.001 |
|  |  | Uninfected vs Propionic | <.001 | <.001 |
|  |  | Uninfected vs KRV+Butyric | <.001 | <.001 |
|  |  | Uninfected vs KRV+Formic | <.001 | <.001 |
|  |  | Uninfected vs KRV+Propionic | <.001 | <.001 |
|  |  | KRV vs KRV+Butyric | 0.105 | 0.206 |
|  |  | KRV vs KRV+Formic | 0.105 | 0.206 |
|  |  | KRV vs KRV+Propionic | 0.105 | 0.206 |
|  |  | KRV+Butyric vs Butyric | 0.168 | 0.285 |
|  |  | KRV+Formic vs Formic | 0.168 | 0.285 |
|  |  | KRV+Propionic vs Propionic | 0.168 | 0.285 |
|  |  | KRV vs Butyric | 1 | 1 |
|  |  | KRV vs Formic | 1 | 1 |
|  |  | KRV vs Propionic | 1 | 1 |
| Marvinbryantia | <.001 | Uninfected vs Butyric | <.001 | <.001 |
|  |  | Uninfected vs Formic | <.001 | <.001 |
|  |  | Uninfected vs Propionic | <.001 | <.001 |
|  |  | KRV vs Butyric | <.001 | 0.004 |
|  |  | KRV vs Formic | <.001 | 0.004 |
|  |  | KRV vs Propionic | <.001 | 0.004 |
|  |  | KRV+Butyric vs Butyric | 0.006 | 0.025 |
|  |  | KRV+Formic vs Formic | 0.006 | 0.025 |
|  |  | KRV+Propionic vs Propionic | 0.006 | 0.025 |
|  |  | Uninfected vs KRV+Butyric | 0.009 | 0.034 |
|  |  | Uninfected vs KRV+Formic | 0.009 | 0.034 |
|  |  | Uninfected vs KRV+Propionic | 0.009 | 0.034 |
|  |  | Uninfected vs KRV | 0.028 | 0.077 |
|  |  | KRV vs KRV+Butyric | 0.479 | 0.638 |
|  |  | KRV vs KRV+Formic | 0.479 | 0.638 |
|  |  | KRV vs KRV+Propionic | 0.479 | 0.638 |
| Pseudomonas | <.001 | KRV vs KRV+Butyric | <.001 | 0.001 |
|  |  | KRV vs KRV+Formic | <.001 | 0.001 |
|  |  | KRV vs KRV+Propionic | <.001 | 0.001 |
|  |  | KRV+Butyric vs Butyric | <.001 | 0.004 |
|  |  | KRV+Formic vs Formic | <.001 | 0.004 |
|  |  | KRV+Propionic vs Propionic | <.001 | 0.004 |
|  |  | Uninfected vs KRV | 0.003 | 0.018 |
|  |  | Uninfected vs Butyric | 0.008 | 0.029 |
|  |  | Uninfected vs Formic | 0.008 | 0.029 |
|  |  | Uninfected vs Propionic | 0.008 | 0.029 |
|  |  | Uninfected vs KRV+Butyric | 0.142 | 0.258 |
|  |  | Uninfected vs KRV+Formic | 0.142 | 0.258 |
|  |  | Uninfected vs KRV+Propionic | 0.142 | 0.258 |
|  |  | KRV vs Butyric | 0.785 | 0.928 |
|  |  | KRV vs Formic | 0.785 | 0.928 |
|  |  | KRV vs Propionic | 0.785 | 0.928 |
| Pasteurellaceae | <.001 | Uninfected vs KRV | <.001 | <.001 |
|  |  | KRV vs KRV+Butyric | <.001 | 0.005 |
|  |  | KRV vs KRV+Formic | <.001 | 0.005 |
|  |  | KRV vs KRV+Propionic | <.001 | 0.005 |
|  |  | Uninfected vs Butyric | 0.001 | 0.009 |
|  |  | Uninfected vs Formic | 0.001 | 0.009 |
|  |  | Uninfected vs Propionic | 0.001 | 0.009 |
|  |  | KRV+Butyric vs Butyric | 0.012 | 0.04 |
|  |  | KRV+Formic vs Formic | 0.012 | 0.04 |
|  |  | KRV+Propionic vs Propionic | 0.012 | 0.04 |
|  |  | Uninfected vs KRV+Butyric | 0.468 | 0.628 |
|  |  | Uninfected vs KRV+Formic | 0.468 | 0.628 |
|  |  | Uninfected vs KRV+Propionic | 0.468 | 0.628 |
|  |  | KRV vs Butyric | 0.545 | 0.705 |
|  |  | KRV vs Formic | 0.545 | 0.705 |
|  |  | KRV vs Propionic | 0.545 | 0.705 |
| Candidate-division-TM7 | <.001 | Uninfected vs KRV | <.001 | <.001 |
|  |  | KRV vs KRV+Butyric | 0.001 | 0.01 |
|  |  | KRV vs KRV+Formic | 0.001 | 0.01 |
|  |  | KRV vs KRV+Propionic | 0.001 | 0.01 |
|  |  | Uninfected vs Butyric | 0.006 | 0.026 |
|  |  | Uninfected vs Formic | 0.006 | 0.026 |
|  |  | Uninfected vs Propionic | 0.006 | 0.026 |
|  |  | KRV vs Butyric | 0.125 | 0.235 |
|  |  | KRV vs Formic | 0.125 | 0.235 |
|  |  | KRV vs Propionic | 0.125 | 0.235 |
|  |  | KRV+Butyric vs Butyric | 0.148 | 0.261 |
|  |  | KRV+Formic vs Formic | 0.148 | 0.261 |
|  |  | KRV+Propionic vs Propionic | 0.148 | 0.261 |
|  |  | Uninfected vs KRV+Butyric | 0.161 | 0.276 |
|  |  | Uninfected vs KRV+Formic | 0.161 | 0.276 |
|  |  | Uninfected vs KRV+Propionic | 0.161 | 0.276 |
| Corynebacterium | <.001 | Uninfected vs Butyric | <.001 | <.001 |
|  |  | Uninfected vs Formic | <.001 | <.001 |
|  |  | Uninfected vs Propionic | <.001 | <.001 |
|  |  | Uninfected vs KRV | <.001 | <.001 |
|  |  | KRV+Butyric vs Butyric | <.001 | 0.003 |
|  |  | KRV+Formic vs Formic | <.001 | 0.003 |
|  |  | KRV+Propionic vs Propionic | <.001 | 0.003 |
|  |  | KRV vs KRV+Butyric | 0.006 | 0.026 |
|  |  | KRV vs KRV+Formic | 0.006 | 0.026 |
|  |  | KRV vs KRV+Propionic | 0.006 | 0.026 |
|  |  | KRV vs Butyric | 0.117 | 0.224 |
|  |  | KRV vs Formic | 0.117 | 0.224 |
|  |  | KRV vs Propionic | 0.117 | 0.224 |
|  |  | Uninfected vs KRV+Butyric | 0.222 | 0.352 |
|  |  | Uninfected vs KRV+Formic | 0.222 | 0.352 |
|  |  | Uninfected vs KRV+Propionic | 0.222 | 0.352 |
| Parabacteroides | <.001 | KRV vs Butyric | <.001 | <.001 |
|  |  | KRV vs Formic | <.001 | <.001 |
|  |  | KRV vs Propionic | <.001 | <.001 |
|  |  | KRV+Butyric vs Butyric | <.001 | 0.003 |
|  |  | KRV+Formic vs Formic | <.001 | 0.003 |
|  |  | KRV+Propionic vs Propionic | <.001 | 0.003 |
|  |  | Uninfected vs Butyric | 0.007 | 0.027 |
|  |  | Uninfected vs Formic | 0.007 | 0.027 |
|  |  | Uninfected vs Propionic | 0.007 | 0.027 |
|  |  | Uninfected vs KRV | 0.019 | 0.055 |
|  |  | Uninfected vs KRV+Butyric | 0.104 | 0.206 |
|  |  | Uninfected vs KRV+Formic | 0.104 | 0.206 |
|  |  | Uninfected vs KRV+Propionic | 0.104 | 0.206 |
|  |  | KRV vs KRV+Butyric | 0.603 | 0.757 |
|  |  | KRV vs KRV+Formic | 0.603 | 0.757 |
|  |  | KRV vs KRV+Propionic | 0.603 | 0.757 |
| Escherichia-Shi | <.001 | KRV vs KRV+Butyric | <.001 | <.001 |
|  |  | KRV vs KRV+Formic | <.001 | <.001 |
|  |  | KRV vs KRV+Propionic | <.001 | <.001 |
|  |  | Uninfected vs KRV | <.001 | 0.005 |
|  |  | KRV+Butyric vs Butyric | 0.013 | 0.04 |
|  |  | KRV+Formic vs Formic | 0.013 | 0.04 |
|  |  | KRV+Propionic vs Propionic | 0.013 | 0.04 |
|  |  | KRV vs Butyric | 0.045 | 0.106 |
|  |  | KRV vs Formic | 0.045 | 0.106 |
|  |  | KRV vs Propionic | 0.045 | 0.106 |
|  |  | Uninfected vs KRV+Butyric | 0.046 | 0.109 |
|  |  | Uninfected vs KRV+Formic | 0.046 | 0.109 |
|  |  | Uninfected vs KRV+Propionic | 0.046 | 0.109 |
|  |  | Uninfected vs Butyric | 0.345 | 0.507 |
|  |  | Uninfected vs Formic | 0.345 | 0.507 |
|  |  | Uninfected vs Propionic | 0.345 | 0.507 |
| Porphyromonadaceae | <.001 | KRV vs Butyric | <.001 | 0.003 |
|  |  | KRV vs Formic | <.001 | 0.003 |
|  |  | KRV vs Propionic | <.001 | 0.003 |
|  |  | KRV vs KRV+Butyric | 0.01 | 0.035 |
|  |  | KRV vs KRV+Formic | 0.01 | 0.035 |
|  |  | KRV vs KRV+Propionic | 0.01 | 0.035 |
|  |  | Uninfected vs Butyric | 0.033 | 0.085 |
|  |  | Uninfected vs Formic | 0.033 | 0.085 |
|  |  | Uninfected vs Propionic | 0.033 | 0.085 |
|  |  | Uninfected vs KRV | 0.037 | 0.094 |
|  |  | KRV+Butyric vs Butyric | 0.177 | 0.295 |
|  |  | KRV+Formic vs Formic | 0.177 | 0.295 |
|  |  | KRV+Propionic vs Propionic | 0.177 | 0.295 |
|  |  | Uninfected vs KRV+Butyric | 0.43 | 0.586 |
|  |  | Uninfected vs KRV+Formic | 0.43 | 0.586 |
|  |  | Uninfected vs KRV+Propionic | 0.43 | 0.586 |
| Bacteroidales | <.001 | Uninfected vs KRV | 0.028 | 0.076 |
|  |  | KRV vs KRV+Butyric | 0.037 | 0.094 |
|  |  | KRV vs KRV+Formic | 0.037 | 0.094 |
|  |  | KRV vs KRV+Propionic | 0.037 | 0.094 |
|  |  | KRV vs Butyric | 0.179 | 0.295 |
|  |  | KRV vs Formic | 0.179 | 0.295 |
|  |  | KRV vs Propionic | 0.179 | 0.295 |
|  |  | KRV+Butyric vs Butyric | 0.581 | 0.736 |
|  |  | KRV+Formic vs Formic | 0.581 | 0.736 |
|  |  | KRV+Propionic vs Propionic | 0.581 | 0.736 |
|  |  | Uninfected vs Butyric | 0.617 | 0.766 |
|  |  | Uninfected vs Formic | 0.617 | 0.766 |
|  |  | Uninfected vs Propionic | 0.617 | 0.766 |
|  |  | Uninfected vs KRV+Butyric | 0.915 | 1 |
|  |  | Uninfected vs KRV+Formic | 0.915 | 1 |
|  |  | Uninfected vs KRV+Propionic | 0.915 | 1 |
| Haemophilus | <.001 | Uninfected vs KRV | <.001 | 0.003 |
|  |  | KRV vs KRV+Butyric | 0.006 | 0.026 |
|  |  | KRV vs KRV+Formic | 0.006 | 0.026 |
|  |  | KRV vs KRV+Propionic | 0.006 | 0.026 |
|  |  | KRV vs Butyric | 0.008 | 0.032 |
|  |  | KRV vs Formic | 0.008 | 0.032 |
|  |  | KRV vs Propionic | 0.008 | 0.032 |
|  |  | Uninfected vs KRV+Butyric | 0.508 | 0.668 |
|  |  | Uninfected vs KRV+Formic | 0.508 | 0.668 |
|  |  | Uninfected vs KRV+Propionic | 0.508 | 0.668 |
|  |  | Uninfected vs Butyric | 0.647 | 0.789 |
|  |  | Uninfected vs Formic | 0.647 | 0.789 |
|  |  | Uninfected vs Propionic | 0.647 | 0.789 |
|  |  | KRV+Butyric vs Butyric | 0.891 | 1 |
|  |  | KRV+Formic vs Formic | 0.891 | 1 |
|  |  | KRV+Propionic vs Propionic | 0.891 | 1 |
| Enterobacteriaceae | <.001 | Uninfected vs KRV | <.001 | <.001 |
|  |  | Uninfected vs Butyric | <.001 | 0.001 |
|  |  | Uninfected vs Formic | <.001 | 0.001 |
|  |  | Uninfected vs Propionic | <.001 | 0.001 |
|  |  | KRV+Butyric vs Butyric | 0.016 | 0.048 |
|  |  | KRV+Formic vs Formic | 0.016 | 0.048 |
|  |  | KRV+Propionic vs Propionic | 0.016 | 0.048 |
|  |  | KRV vs KRV+Butyric | 0.039 | 0.097 |
|  |  | KRV vs KRV+Formic | 0.039 | 0.097 |
|  |  | KRV vs KRV+Propionic | 0.039 | 0.097 |
|  |  | Uninfected vs KRV+Butyric | 0.074 | 0.156 |
|  |  | Uninfected vs KRV+Formic | 0.074 | 0.156 |
|  |  | Uninfected vs KRV+Propionic | 0.074 | 0.156 |
|  |  | KRV vs Butyric | 0.437 | 0.594 |
|  |  | KRV vs Formic | 0.437 | 0.594 |
|  |  | KRV vs Propionic | 0.437 | 0.594 |
| Turicibacter | <.001 | Uninfected vs KRV+Butyric | 0.003 | 0.018 |
|  |  | Uninfected vs KRV+Formic | 0.003 | 0.018 |
|  |  | Uninfected vs KRV+Propionic | 0.003 | 0.018 |
|  |  | KRV vs KRV+Butyric | 0.005 | 0.024 |
|  |  | KRV vs KRV+Formic | 0.005 | 0.024 |
|  |  | KRV vs KRV+Propionic | 0.005 | 0.024 |
|  |  | Uninfected vs Butyric | 0.17 | 0.285 |
|  |  | Uninfected vs Formic | 0.17 | 0.285 |
|  |  | Uninfected vs Propionic | 0.17 | 0.285 |
|  |  | KRV+Butyric vs Butyric | 0.183 | 0.3 |
|  |  | KRV+Formic vs Formic | 0.183 | 0.3 |
|  |  | KRV+Propionic vs Propionic | 0.183 | 0.3 |
|  |  | KRV vs Butyric | 0.224 | 0.354 |
|  |  | KRV vs Formic | 0.224 | 0.354 |
|  |  | KRV vs Propionic | 0.224 | 0.354 |
|  |  | Uninfected vs KRV | 0.844 | 0.982 |
| Gemmobacter | <.001 | KRV vs KRV+Butyric | <.001 | <.001 |
|  |  | KRV vs KRV+Formic | <.001 | <.001 |
|  |  | KRV vs KRV+Propionic | <.001 | <.001 |
|  |  | KRV+Butyric vs Butyric | <.001 | <.001 |
|  |  | KRV+Formic vs Formic | <.001 | <.001 |
|  |  | KRV+Propionic vs Propionic | <.001 | <.001 |
|  |  | Uninfected vs KRV+Butyric | <.001 | 0.004 |
|  |  | Uninfected vs KRV+Formic | <.001 | 0.004 |
|  |  | Uninfected vs KRV+Propionic | <.001 | 0.004 |
|  |  | Uninfected vs KRV | 0.055 | 0.125 |
|  |  | Uninfected vs Butyric | 0.114 | 0.222 |
|  |  | Uninfected vs Formic | 0.114 | 0.222 |
|  |  | Uninfected vs Propionic | 0.114 | 0.222 |
|  |  | KRV vs Butyric | 1 | 1 |
|  |  | KRV vs Formic | 1 | 1 |
|  |  | KRV vs Propionic | 1 | 1 |
| Enterobacter | <.001 | KRV vs KRV+Butyric | <.001 | <.001 |
|  |  | KRV vs KRV+Formic | <.001 | <.001 |
|  |  | KRV vs KRV+Propionic | <.001 | <.001 |
|  |  | Uninfected vs KRV | <.001 | 0.007 |
|  |  | KRV+Butyric vs Butyric | 0.015 | 0.046 |
|  |  | KRV+Formic vs Formic | 0.015 | 0.046 |
|  |  | KRV+Propionic vs Propionic | 0.015 | 0.046 |
|  |  | Uninfected vs Butyric | 0.127 | 0.237 |
|  |  | Uninfected vs Formic | 0.127 | 0.237 |
|  |  | Uninfected vs Propionic | 0.127 | 0.237 |
|  |  | KRV vs Butyric | 0.169 | 0.285 |
|  |  | KRV vs Formic | 0.169 | 0.285 |
|  |  | KRV vs Propionic | 0.169 | 0.285 |
|  |  | Uninfected vs KRV+Butyric | 0.195 | 0.318 |
|  |  | Uninfected vs KRV+Formic | 0.195 | 0.318 |
|  |  | Uninfected vs KRV+Propionic | 0.195 | 0.318 |
| Christensenellaceae | <.001 | KRV vs Butyric | 0.004 | 0.019 |
|  |  | KRV vs Formic | 0.004 | 0.019 |
|  |  | KRV vs Propionic | 0.004 | 0.019 |
|  |  | KRV+Butyric vs Butyric | 0.004 | 0.019 |
|  |  | KRV+Formic vs Formic | 0.004 | 0.019 |
|  |  | KRV+Propionic vs Propionic | 0.004 | 0.019 |
|  |  | Uninfected vs Butyric | 0.013 | 0.041 |
|  |  | Uninfected vs Formic | 0.013 | 0.041 |
|  |  | Uninfected vs Propionic | 0.013 | 0.041 |
|  |  | Uninfected vs KRV+Butyric | 0.451 | 0.61 |
|  |  | Uninfected vs KRV+Formic | 0.451 | 0.61 |
|  |  | Uninfected vs KRV+Propionic | 0.451 | 0.61 |
|  |  | Uninfected vs KRV | 0.567 | 0.729 |
|  |  | KRV vs KRV+Butyric | 0.808 | 0.951 |
|  |  | KRV vs KRV+Formic | 0.808 | 0.951 |
|  |  | KRV vs KRV+Propionic | 0.808 | 0.951 |
| Lactobacillales | <.001 | Uninfected vs KRV+Butyric | <.001 | 0.007 |
|  |  | Uninfected vs KRV+Formic | <.001 | 0.007 |
|  |  | Uninfected vs KRV+Propionic | <.001 | 0.007 |
|  |  | Uninfected vs KRV | 0.001 | 0.009 |
|  |  | KRV+Butyric vs Butyric | 0.029 | 0.077 |
|  |  | KRV+Formic vs Formic | 0.029 | 0.077 |
|  |  | KRV+Propionic vs Propionic | 0.029 | 0.077 |
|  |  | KRV vs Butyric | 0.052 | 0.119 |
|  |  | KRV vs Formic | 0.052 | 0.119 |
|  |  | KRV vs Propionic | 0.052 | 0.119 |
|  |  | Uninfected vs Butyric | 0.406 | 0.562 |
|  |  | Uninfected vs Formic | 0.406 | 0.562 |
|  |  | Uninfected vs Propionic | 0.406 | 0.562 |
|  |  | KRV vs KRV+Butyric | 0.62 | 0.766 |
|  |  | KRV vs KRV+Formic | 0.62 | 0.766 |
|  |  | KRV vs KRV+Propionic | 0.62 | 0.766 |
| Proteus | <.001 | Uninfected vs KRV+Butyric | <.001 | <.001 |
|  |  | Uninfected vs KRV+Formic | <.001 | <.001 |
|  |  | Uninfected vs KRV+Propionic | <.001 | <.001 |
|  |  | Uninfected vs Butyric | <.001 | <.001 |
|  |  | Uninfected vs Formic | <.001 | <.001 |
|  |  | Uninfected vs Propionic | <.001 | <.001 |
|  |  | Uninfected vs KRV | <.001 | 0.007 |
|  |  | KRV vs KRV+Butyric | 0.084 | 0.174 |
|  |  | KRV vs KRV+Formic | 0.084 | 0.174 |
|  |  | KRV vs KRV+Propionic | 0.084 | 0.174 |
|  |  | KRV vs Butyric | 0.113 | 0.221 |
|  |  | KRV vs Formic | 0.113 | 0.221 |
|  |  | KRV vs Propionic | 0.113 | 0.221 |
|  |  | KRV+Butyric vs Butyric | 1 | 1 |
|  |  | KRV+Formic vs Formic | 1 | 1 |
|  |  | KRV+Propionic vs Propionic | 1 | 1 |
| Acetitomaculum | <.001 | KRV vs Butyric | 0.002 | 0.011 |
|  |  | KRV vs Formic | 0.002 | 0.011 |
|  |  | KRV vs Propionic | 0.002 | 0.011 |
|  |  | KRV+Butyric vs Butyric | 0.003 | 0.016 |
|  |  | KRV+Formic vs Formic | 0.003 | 0.016 |
|  |  | KRV+Propionic vs Propionic | 0.003 | 0.016 |
|  |  | Uninfected vs Butyric | 0.03 | 0.078 |
|  |  | Uninfected vs Formic | 0.03 | 0.078 |
|  |  | Uninfected vs Propionic | 0.03 | 0.078 |
|  |  | Uninfected vs KRV | 0.169 | 0.285 |
|  |  | Uninfected vs KRV+Butyric | 0.203 | 0.328 |
|  |  | Uninfected vs KRV+Formic | 0.203 | 0.328 |
|  |  | Uninfected vs KRV+Propionic | 0.203 | 0.328 |
|  |  | KRV vs KRV+Butyric | 0.967 | 1 |
|  |  | KRV vs KRV+Formic | 0.967 | 1 |
|  |  | KRV vs KRV+Propionic | 0.967 | 1 |
| Staphylococcus | <.001 | Uninfected vs Butyric | <.001 | 0.003 |
|  |  | Uninfected vs Formic | <.001 | 0.003 |
|  |  | Uninfected vs Propionic | <.001 | 0.003 |
|  |  | KRV vs Butyric | 0.012 | 0.04 |
|  |  | KRV vs Formic | 0.012 | 0.04 |
|  |  | KRV vs Propionic | 0.012 | 0.04 |
|  |  | Uninfected vs KRV+Butyric | 0.012 | 0.04 |
|  |  | Uninfected vs KRV+Formic | 0.012 | 0.04 |
|  |  | Uninfected vs KRV+Propionic | 0.012 | 0.04 |
|  |  | Uninfected vs KRV | 0.106 | 0.207 |
|  |  | KRV+Butyric vs Butyric | 0.149 | 0.262 |
|  |  | KRV+Formic vs Formic | 0.149 | 0.262 |
|  |  | KRV+Propionic vs Propionic | 0.149 | 0.262 |
|  |  | KRV vs KRV+Butyric | 0.253 | 0.391 |
|  |  | KRV vs KRV+Formic | 0.253 | 0.391 |
|  |  | KRV vs KRV+Propionic | 0.253 | 0.391 |
| Citrobacter | <.001 | Uninfected vs KRV | <.001 | <.001 |
|  |  | KRV vs Butyric | <.001 | 0.004 |
|  |  | KRV vs Formic | <.001 | 0.004 |
|  |  | KRV vs Propionic | <.001 | 0.004 |
|  |  | KRV vs KRV+Butyric | 0.019 | 0.055 |
|  |  | KRV vs KRV+Formic | 0.019 | 0.055 |
|  |  | KRV vs KRV+Propionic | 0.019 | 0.055 |
|  |  | Uninfected vs KRV+Butyric | 0.086 | 0.174 |
|  |  | Uninfected vs KRV+Formic | 0.086 | 0.174 |
|  |  | Uninfected vs KRV+Propionic | 0.086 | 0.174 |
|  |  | KRV+Butyric vs Butyric | 0.144 | 0.259 |
|  |  | KRV+Formic vs Formic | 0.144 | 0.259 |
|  |  | KRV+Propionic vs Propionic | 0.144 | 0.259 |
|  |  | Uninfected vs Butyric | 1 | 1 |
|  |  | Uninfected vs Formic | 1 | 1 |
|  |  | Uninfected vs Propionic | 1 | 1 |
| Comamonas | <.001 | Uninfected vs KRV | 0.292 | 0.445 |
|  |  | KRV vs KRV+Butyric | 0.345 | 0.507 |
|  |  | KRV vs KRV+Formic | 0.345 | 0.507 |
|  |  | KRV vs KRV+Propionic | 0.345 | 0.507 |
|  |  | KRV vs Butyric | 0.388 | 0.548 |
|  |  | KRV vs Formic | 0.388 | 0.548 |
|  |  | KRV vs Propionic | 0.388 | 0.548 |
|  |  | Uninfected vs KRV+Butyric | 1 | 1 |
|  |  | Uninfected vs Butyric | 1 | 1 |
|  |  | KRV+Butyric vs Butyric | 1 | 1 |
|  |  | Uninfected vs KRV+Formic | 1 | 1 |
|  |  | Uninfected vs Formic | 1 | 1 |
|  |  | KRV+Formic vs Formic | 1 | 1 |
|  |  | Uninfected vs KRV+Propionic | 1 | 1 |
|  |  | Uninfected vs Propionic | 1 | 1 |
|  |  | KRV+Propionic vs Propionic | 1 | 1 |
| Bacteroides | <.001 | Uninfected vs KRV | <.001 | 0.005 |
|  |  | KRV vs Butyric | 0.017 | 0.049 |
|  |  | KRV vs Formic | 0.017 | 0.049 |
|  |  | KRV vs Propionic | 0.017 | 0.049 |
|  |  | KRV vs KRV+Butyric | 0.017 | 0.049 |
|  |  | KRV vs KRV+Formic | 0.017 | 0.049 |
|  |  | KRV vs KRV+Propionic | 0.017 | 0.049 |
|  |  | Uninfected vs KRV+Butyric | 0.408 | 0.563 |
|  |  | Uninfected vs KRV+Formic | 0.408 | 0.563 |
|  |  | Uninfected vs KRV+Propionic | 0.408 | 0.563 |
|  |  | Uninfected vs Butyric | 0.591 | 0.747 |
|  |  | Uninfected vs Formic | 0.591 | 0.747 |
|  |  | Uninfected vs Propionic | 0.591 | 0.747 |
|  |  | KRV+Butyric vs Butyric | 0.839 | 0.976 |
|  |  | KRV+Formic vs Formic | 0.839 | 0.976 |
|  |  | KRV+Propionic vs Propionic | 0.839 | 0.976 |
| Stenotrophomonas | <.001 | KRV vs Butyric | 0.002 | 0.011 |
|  |  | KRV vs Formic | 0.002 | 0.011 |
|  |  | KRV vs Propionic | 0.002 | 0.011 |
|  |  | KRV+Butyric vs Butyric | 0.003 | 0.017 |
|  |  | KRV+Formic vs Formic | 0.003 | 0.017 |
|  |  | KRV+Propionic vs Propionic | 0.003 | 0.017 |
|  |  | Uninfected vs KRV | 0.01 | 0.036 |
|  |  | Uninfected vs KRV+Butyric | 0.02 | 0.056 |
|  |  | Uninfected vs KRV+Formic | 0.02 | 0.056 |
|  |  | Uninfected vs KRV+Propionic | 0.02 | 0.056 |
|  |  | Uninfected vs Butyric | 0.24 | 0.376 |
|  |  | Uninfected vs Formic | 0.24 | 0.376 |
|  |  | Uninfected vs Propionic | 0.24 | 0.376 |
|  |  | KRV vs KRV+Butyric | 1 | 1 |
|  |  | KRV vs KRV+Formic | 1 | 1 |
|  |  | KRV vs KRV+Propionic | 1 | 1 |
| Streptococcus | <.001 | KRV+Butyric vs Butyric | <.001 | 0.003 |
|  |  | KRV+Formic vs Formic | <.001 | 0.003 |
|  |  | KRV+Propionic vs Propionic | <.001 | 0.003 |
|  |  | Uninfected vs Butyric | <.001 | 0.005 |
|  |  | Uninfected vs Formic | <.001 | 0.005 |
|  |  | Uninfected vs Propionic | <.001 | 0.005 |
|  |  | KRV vs Butyric | 0.005 | 0.022 |
|  |  | KRV vs Formic | 0.005 | 0.022 |
|  |  | KRV vs Propionic | 0.005 | 0.022 |
|  |  | KRV vs KRV+Butyric | 0.152 | 0.266 |
|  |  | KRV vs KRV+Formic | 0.152 | 0.266 |
|  |  | KRV vs KRV+Propionic | 0.152 | 0.266 |
|  |  | Uninfected vs KRV | 0.374 | 0.532 |
|  |  | Uninfected vs KRV+Butyric | 0.515 | 0.67 |
|  |  | Uninfected vs KRV+Formic | 0.515 | 0.67 |
|  |  | Uninfected vs KRV+Propionic | 0.515 | 0.67 |
| Betaproteobacteria | <.001 | Uninfected vs KRV | <.001 | 0.003 |
|  |  | KRV vs KRV+Butyric | <.001 | 0.007 |
|  |  | KRV vs KRV+Formic | <.001 | 0.007 |
|  |  | KRV vs KRV+Propionic | <.001 | 0.007 |
|  |  | KRV vs Butyric | 0.002 | 0.013 |
|  |  | KRV vs Formic | 0.002 | 0.013 |
|  |  | KRV vs Propionic | 0.002 | 0.013 |
|  |  | Uninfected vs KRV+Butyric | 1 | 1 |
|  |  | Uninfected vs Butyric | 1 | 1 |
|  |  | KRV+Butyric vs Butyric | 1 | 1 |
|  |  | Uninfected vs KRV+Formic | 1 | 1 |
|  |  | Uninfected vs Formic | 1 | 1 |
|  |  | KRV+Formic vs Formic | 1 | 1 |
|  |  | Uninfected vs KRV+Propionic | 1 | 1 |
|  |  | Uninfected vs Propionic | 1 | 1 |
|  |  | KRV+Propionic vs Propionic | 1 | 1 |
| Caldicoprobacter | <.001 | Uninfected vs Butyric | 0.01 | 0.036 |
|  |  | Uninfected vs Formic | 0.01 | 0.036 |
|  |  | Uninfected vs Propionic | 0.01 | 0.036 |
|  |  | KRV+Butyric vs Butyric | 0.011 | 0.037 |
|  |  | KRV+Formic vs Formic | 0.011 | 0.037 |
|  |  | KRV+Propionic vs Propionic | 0.011 | 0.037 |
|  |  | KRV vs Butyric | 0.045 | 0.106 |
|  |  | KRV vs Formic | 0.045 | 0.106 |
|  |  | KRV vs Propionic | 0.045 | 0.106 |
|  |  | KRV vs KRV+Butyric | 0.368 | 0.528 |
|  |  | KRV vs KRV+Formic | 0.368 | 0.528 |
|  |  | KRV vs KRV+Propionic | 0.368 | 0.528 |
|  |  | Uninfected vs KRV | 0.449 | 0.61 |
|  |  | Uninfected vs KRV+Butyric | 0.821 | 0.959 |
|  |  | Uninfected vs KRV+Formic | 0.821 | 0.959 |
|  |  | Uninfected vs KRV+Propionic | 0.821 | 0.959 |
| Blautia | <.001 | Uninfected vs KRV | 0.001 | 0.009 |
|  |  | KRV vs Butyric | 0.002 | 0.01 |
|  |  | KRV vs Formic | 0.002 | 0.01 |
|  |  | KRV vs Propionic | 0.002 | 0.01 |
|  |  | KRV+Butyric vs Butyric | 0.05 | 0.116 |
|  |  | KRV+Formic vs Formic | 0.05 | 0.116 |
|  |  | KRV+Propionic vs Propionic | 0.05 | 0.116 |
|  |  | Uninfected vs KRV+Butyric | 0.089 | 0.18 |
|  |  | Uninfected vs KRV+Formic | 0.089 | 0.18 |
|  |  | Uninfected vs KRV+Propionic | 0.089 | 0.18 |
|  |  | KRV vs KRV+Butyric | 0.181 | 0.299 |
|  |  | KRV vs KRV+Formic | 0.181 | 0.299 |
|  |  | KRV vs KRV+Propionic | 0.181 | 0.299 |
|  |  | Uninfected vs Butyric | 0.556 | 0.716 |
|  |  | Uninfected vs Formic | 0.556 | 0.716 |
|  |  | Uninfected vs Propionic | 0.556 | 0.716 |
| Family-XIII-Incertae-Sedis | <.001 | KRV vs Butyric | 0.002 | 0.011 |
|  |  | KRV vs Formic | 0.002 | 0.011 |
|  |  | KRV vs Propionic | 0.002 | 0.011 |
|  |  | Uninfected vs Butyric | 0.016 | 0.048 |
|  |  | Uninfected vs Formic | 0.016 | 0.048 |
|  |  | Uninfected vs Propionic | 0.016 | 0.048 |
|  |  | KRV+Butyric vs Butyric | 0.029 | 0.078 |
|  |  | KRV+Formic vs Formic | 0.029 | 0.078 |
|  |  | KRV+Propionic vs Propionic | 0.029 | 0.078 |
|  |  | Uninfected vs KRV | 0.294 | 0.447 |
|  |  | KRV vs KRV+Butyric | 0.303 | 0.458 |
|  |  | KRV vs KRV+Formic | 0.303 | 0.458 |
|  |  | KRV vs KRV+Propionic | 0.303 | 0.458 |
|  |  | Uninfected vs KRV+Butyric | 0.927 | 1 |
|  |  | Uninfected vs KRV+Formic | 0.927 | 1 |
|  |  | Uninfected vs KRV+Propionic | 0.927 | 1 |
| Bifidobacterium | <.001 | KRV vs KRV+Butyric | 0.054 | 0.123 |
|  |  | KRV vs KRV+Formic | 0.054 | 0.123 |
|  |  | KRV vs KRV+Propionic | 0.054 | 0.123 |
|  |  | Uninfected vs KRV+Butyric | 0.064 | 0.14 |
|  |  | Uninfected vs KRV+Formic | 0.064 | 0.14 |
|  |  | Uninfected vs KRV+Propionic | 0.064 | 0.14 |
|  |  | KRV+Butyric vs Butyric | 0.136 | 0.25 |
|  |  | KRV+Formic vs Formic | 0.136 | 0.25 |
|  |  | KRV+Propionic vs Propionic | 0.136 | 0.25 |
|  |  | KRV vs Butyric | 0.867 | 1 |
|  |  | KRV vs Formic | 0.867 | 1 |
|  |  | KRV vs Propionic | 0.867 | 1 |
|  |  | Uninfected vs Butyric | 0.924 | 1 |
|  |  | Uninfected vs Formic | 0.924 | 1 |
|  |  | Uninfected vs Propionic | 0.924 | 1 |
|  |  | Uninfected vs KRV | 0.93 | 1 |
| Clostridium | 0.001 | Uninfected vs KRV+Butyric | 0.003 | 0.017 |
|  |  | Uninfected vs KRV+Formic | 0.003 | 0.017 |
|  |  | Uninfected vs KRV+Propionic | 0.003 | 0.017 |
|  |  | KRV vs KRV+Butyric | 0.019 | 0.055 |
|  |  | KRV vs KRV+Formic | 0.019 | 0.055 |
|  |  | KRV vs KRV+Propionic | 0.019 | 0.055 |
|  |  | KRV+Butyric vs Butyric | 0.155 | 0.268 |
|  |  | KRV+Formic vs Formic | 0.155 | 0.268 |
|  |  | KRV+Propionic vs Propionic | 0.155 | 0.268 |
|  |  | Uninfected vs Butyric | 0.191 | 0.312 |
|  |  | Uninfected vs Formic | 0.191 | 0.312 |
|  |  | Uninfected vs Propionic | 0.191 | 0.312 |
|  |  | Uninfected vs KRV | 0.42 | 0.578 |
|  |  | KRV vs Butyric | 0.51 | 0.669 |
|  |  | KRV vs Formic | 0.51 | 0.669 |
|  |  | KRV vs Propionic | 0.51 | 0.669 |
| Coriobacteriaceae | 0.001 | KRV vs KRV+Butyric | 0.002 | 0.012 |
|  |  | KRV vs KRV+Formic | 0.002 | 0.012 |
|  |  | KRV vs KRV+Propionic | 0.002 | 0.012 |
|  |  | KRV vs Butyric | 0.002 | 0.013 |
|  |  | KRV vs Formic | 0.002 | 0.013 |
|  |  | KRV vs Propionic | 0.002 | 0.013 |
|  |  | Uninfected vs KRV | 0.057 | 0.128 |
|  |  | Uninfected vs Butyric | 0.102 | 0.204 |
|  |  | Uninfected vs Formic | 0.102 | 0.204 |
|  |  | Uninfected vs Propionic | 0.102 | 0.204 |
|  |  | Uninfected vs KRV+Butyric | 0.126 | 0.235 |
|  |  | Uninfected vs KRV+Formic | 0.126 | 0.235 |
|  |  | Uninfected vs KRV+Propionic | 0.126 | 0.235 |
|  |  | KRV+Butyric vs Butyric | 0.819 | 0.959 |
|  |  | KRV+Formic vs Formic | 0.819 | 0.959 |
|  |  | KRV+Propionic vs Propionic | 0.819 | 0.959 |
| Anaeroplasma | 0.001 | Uninfected vs KRV | 0.048 | 0.113 |
|  |  | KRV vs Butyric | 0.221 | 0.352 |
|  |  | KRV vs Formic | 0.221 | 0.352 |
|  |  | KRV vs Propionic | 0.221 | 0.352 |
|  |  | KRV vs KRV+Butyric | 0.31 | 0.466 |
|  |  | KRV vs KRV+Formic | 0.31 | 0.466 |
|  |  | KRV vs KRV+Propionic | 0.31 | 0.466 |
|  |  | Uninfected vs KRV+Butyric | 0.43 | 0.586 |
|  |  | Uninfected vs KRV+Formic | 0.43 | 0.586 |
|  |  | Uninfected vs KRV+Propionic | 0.43 | 0.586 |
|  |  | Uninfected vs Butyric | 0.674 | 0.808 |
|  |  | Uninfected vs Formic | 0.674 | 0.808 |
|  |  | Uninfected vs Propionic | 0.674 | 0.808 |
|  |  | KRV+Butyric vs Butyric | 0.779 | 0.925 |
|  |  | KRV+Formic vs Formic | 0.779 | 0.925 |
|  |  | KRV+Propionic vs Propionic | 0.779 | 0.925 |
| Peptococcaceae | 0.001 | Uninfected vs KRV | 0.003 | 0.016 |
|  |  | Uninfected vs KRV+Butyric | 0.007 | 0.027 |
|  |  | Uninfected vs KRV+Formic | 0.007 | 0.027 |
|  |  | Uninfected vs KRV+Propionic | 0.007 | 0.027 |
|  |  | KRV vs Butyric | 0.042 | 0.1 |
|  |  | KRV vs Formic | 0.042 | 0.1 |
|  |  | KRV vs Propionic | 0.042 | 0.1 |
|  |  | KRV+Butyric vs Butyric | 0.056 | 0.126 |
|  |  | KRV+Formic vs Formic | 0.056 | 0.126 |
|  |  | KRV+Propionic vs Propionic | 0.056 | 0.126 |
|  |  | Uninfected vs Butyric | 0.625 | 0.769 |
|  |  | Uninfected vs Formic | 0.625 | 0.769 |
|  |  | Uninfected vs Propionic | 0.625 | 0.769 |
|  |  | KRV vs KRV+Butyric | 0.98 | 1 |
|  |  | KRV vs KRV+Formic | 0.98 | 1 |
|  |  | KRV vs KRV+Propionic | 0.98 | 1 |
| Butyricimonas | 0.001 | Uninfected vs KRV | 0.001 | 0.008 |
|  |  | Uninfected vs Butyric | 0.009 | 0.035 |
|  |  | Uninfected vs Formic | 0.009 | 0.035 |
|  |  | Uninfected vs Propionic | 0.009 | 0.035 |
|  |  | KRV vs KRV+Butyric | 0.012 | 0.039 |
|  |  | KRV vs KRV+Formic | 0.012 | 0.039 |
|  |  | KRV vs KRV+Propionic | 0.012 | 0.039 |
|  |  | KRV+Butyric vs Butyric | 0.041 | 0.1 |
|  |  | KRV+Formic vs Formic | 0.041 | 0.1 |
|  |  | KRV+Propionic vs Propionic | 0.041 | 0.1 |
|  |  | Uninfected vs KRV+Butyric | 0.619 | 0.766 |
|  |  | Uninfected vs KRV+Formic | 0.619 | 0.766 |
|  |  | Uninfected vs KRV+Propionic | 0.619 | 0.766 |
|  |  | KRV vs Butyric | 0.889 | 1 |
|  |  | KRV vs Formic | 0.889 | 1 |
|  |  | KRV vs Propionic | 0.889 | 1 |
| Candidatus-Arthromitus | 0.002 | KRV vs Butyric | 0.011 | 0.038 |
|  |  | KRV vs Formic | 0.011 | 0.038 |
|  |  | KRV vs Propionic | 0.011 | 0.038 |
|  |  | Uninfected vs KRV | 0.037 | 0.093 |
|  |  | KRV+Butyric vs Butyric | 0.104 | 0.206 |
|  |  | KRV+Formic vs Formic | 0.104 | 0.206 |
|  |  | KRV+Propionic vs Propionic | 0.104 | 0.206 |
|  |  | Uninfected vs KRV+Butyric | 0.332 | 0.498 |
|  |  | Uninfected vs KRV+Formic | 0.332 | 0.498 |
|  |  | Uninfected vs KRV+Propionic | 0.332 | 0.498 |
|  |  | KRV vs KRV+Butyric | 0.345 | 0.507 |
|  |  | KRV vs KRV+Formic | 0.345 | 0.507 |
|  |  | KRV vs KRV+Propionic | 0.345 | 0.507 |
|  |  | Uninfected vs Butyric | 0.373 | 0.532 |
|  |  | Uninfected vs Formic | 0.373 | 0.532 |
|  |  | Uninfected vs Propionic | 0.373 | 0.532 |
| Paraprevotella | 0.002 | KRV vs KRV+Butyric | <.001 | 0.005 |
|  |  | KRV vs KRV+Formic | <.001 | 0.005 |
|  |  | KRV vs KRV+Propionic | <.001 | 0.005 |
|  |  | KRV+Butyric vs Butyric | 0.022 | 0.062 |
|  |  | KRV+Formic vs Formic | 0.022 | 0.062 |
|  |  | KRV+Propionic vs Propionic | 0.022 | 0.062 |
|  |  | Uninfected vs KRV+Butyric | 0.029 | 0.078 |
|  |  | Uninfected vs KRV+Formic | 0.029 | 0.078 |
|  |  | Uninfected vs KRV+Propionic | 0.029 | 0.078 |
|  |  | Uninfected vs KRV | 0.102 | 0.204 |
|  |  | KRV vs Butyric | 0.392 | 0.55 |
|  |  | KRV vs Formic | 0.392 | 0.55 |
|  |  | KRV vs Propionic | 0.392 | 0.55 |
|  |  | Uninfected vs Butyric | 0.615 | 0.766 |
|  |  | Uninfected vs Formic | 0.615 | 0.766 |
|  |  | Uninfected vs Propionic | 0.615 | 0.766 |
| Roseburia | 0.004 | Uninfected vs KRV+Butyric | 0.005 | 0.023 |
|  |  | Uninfected vs KRV+Formic | 0.005 | 0.023 |
|  |  | Uninfected vs KRV+Propionic | 0.005 | 0.023 |
|  |  | Uninfected vs KRV | 0.03 | 0.079 |
|  |  | Uninfected vs Butyric | 0.042 | 0.1 |
|  |  | Uninfected vs Formic | 0.042 | 0.1 |
|  |  | Uninfected vs Propionic | 0.042 | 0.1 |
|  |  | KRV vs KRV+Butyric | 0.338 | 0.504 |
|  |  | KRV vs KRV+Formic | 0.338 | 0.504 |
|  |  | KRV vs KRV+Propionic | 0.338 | 0.504 |
|  |  | KRV+Butyric vs Butyric | 0.573 | 0.732 |
|  |  | KRV+Formic vs Formic | 0.573 | 0.732 |
|  |  | KRV+Propionic vs Propionic | 0.573 | 0.732 |
|  |  | KRV vs Butyric | 0.787 | 0.928 |
|  |  | KRV vs Formic | 0.787 | 0.928 |
|  |  | KRV vs Propionic | 0.787 | 0.928 |
| Oscillibacter | 0.004 | KRV vs Butyric | 0.035 | 0.089 |
|  |  | KRV vs Formic | 0.035 | 0.089 |
|  |  | KRV vs Propionic | 0.035 | 0.089 |
|  |  | Uninfected vs Butyric | 0.066 | 0.142 |
|  |  | Uninfected vs Formic | 0.066 | 0.142 |
|  |  | Uninfected vs Propionic | 0.066 | 0.142 |
|  |  | KRV vs KRV+Butyric | 0.085 | 0.174 |
|  |  | KRV vs KRV+Formic | 0.085 | 0.174 |
|  |  | KRV vs KRV+Propionic | 0.085 | 0.174 |
|  |  | Uninfected vs KRV+Butyric | 0.157 | 0.27 |
|  |  | Uninfected vs KRV+Formic | 0.157 | 0.27 |
|  |  | Uninfected vs KRV+Propionic | 0.157 | 0.27 |
|  |  | KRV+Butyric vs Butyric | 0.597 | 0.752 |
|  |  | KRV+Formic vs Formic | 0.597 | 0.752 |
|  |  | KRV+Propionic vs Propionic | 0.597 | 0.752 |
|  |  | Uninfected vs KRV | 0.721 | 0.862 |
| Rikenella | 0.005 | KRV+Butyric vs Butyric | 0.052 | 0.119 |
|  |  | KRV+Formic vs Formic | 0.052 | 0.119 |
|  |  | KRV+Propionic vs Propionic | 0.052 | 0.119 |
|  |  | Uninfected vs Butyric | 0.064 | 0.14 |
|  |  | Uninfected vs Formic | 0.064 | 0.14 |
|  |  | Uninfected vs Propionic | 0.064 | 0.14 |
|  |  | KRV vs Butyric | 0.119 | 0.227 |
|  |  | KRV vs Formic | 0.119 | 0.227 |
|  |  | KRV vs Propionic | 0.119 | 0.227 |
|  |  | KRV vs KRV+Butyric | 0.534 | 0.693 |
|  |  | KRV vs KRV+Formic | 0.534 | 0.693 |
|  |  | KRV vs KRV+Propionic | 0.534 | 0.693 |
|  |  | Uninfected vs KRV | 0.702 | 0.841 |
|  |  | Uninfected vs KRV+Butyric | 0.779 | 0.925 |
|  |  | Uninfected vs KRV+Formic | 0.779 | 0.925 |
|  |  | Uninfected vs KRV+Propionic | 0.779 | 0.925 |
| Proteobacteria | 0.006 | KRV vs KRV+Butyric | 0.012 | 0.04 |
|  |  | KRV vs KRV+Formic | 0.012 | 0.04 |
|  |  | KRV vs KRV+Propionic | 0.012 | 0.04 |
|  |  | KRV+Butyric vs Butyric | 0.025 | 0.069 |
|  |  | KRV+Formic vs Formic | 0.025 | 0.069 |
|  |  | KRV+Propionic vs Propionic | 0.025 | 0.069 |
|  |  | Uninfected vs KRV+Butyric | 0.12 | 0.227 |
|  |  | Uninfected vs KRV+Formic | 0.12 | 0.227 |
|  |  | Uninfected vs KRV+Propionic | 0.12 | 0.227 |
|  |  | Uninfected vs KRV | 0.252 | 0.391 |
|  |  | Uninfected vs Butyric | 0.29 | 0.443 |
|  |  | Uninfected vs Formic | 0.29 | 0.443 |
|  |  | Uninfected vs Propionic | 0.29 | 0.443 |
|  |  | KRV vs Butyric | 0.903 | 1 |
|  |  | KRV vs Formic | 0.903 | 1 |
|  |  | KRV vs Propionic | 0.903 | 1 |
| Ruminococcus | 0.006 | KRV+Butyric vs Butyric | 0.002 | 0.011 |
|  |  | KRV+Formic vs Formic | 0.002 | 0.011 |
|  |  | KRV+Propionic vs Propionic | 0.002 | 0.011 |
|  |  | KRV vs Butyric | 0.004 | 0.018 |
|  |  | KRV vs Formic | 0.004 | 0.018 |
|  |  | KRV vs Propionic | 0.004 | 0.018 |
|  |  | Uninfected vs KRV+Butyric | 0.01 | 0.035 |
|  |  | Uninfected vs KRV+Formic | 0.01 | 0.035 |
|  |  | Uninfected vs KRV+Propionic | 0.01 | 0.035 |
|  |  | Uninfected vs KRV | 0.02 | 0.057 |
|  |  | Uninfected vs Butyric | 0.262 | 0.404 |
|  |  | Uninfected vs Formic | 0.262 | 0.404 |
|  |  | Uninfected vs Propionic | 0.262 | 0.404 |
|  |  | KRV vs KRV+Butyric | 0.577 | 0.734 |
|  |  | KRV vs KRV+Formic | 0.577 | 0.734 |
|  |  | KRV vs KRV+Propionic | 0.577 | 0.734 |
| Anaerostipes | 0.007 | Uninfected vs KRV | <.001 | 0.002 |
|  |  | Uninfected vs Butyric | 0.016 | 0.048 |
|  |  | Uninfected vs Formic | 0.016 | 0.048 |
|  |  | Uninfected vs Propionic | 0.016 | 0.048 |
|  |  | KRV vs KRV+Butyric | 0.029 | 0.078 |
|  |  | KRV vs KRV+Formic | 0.029 | 0.078 |
|  |  | KRV vs KRV+Propionic | 0.029 | 0.078 |
|  |  | Uninfected vs KRV+Butyric | 0.142 | 0.258 |
|  |  | Uninfected vs KRV+Formic | 0.142 | 0.258 |
|  |  | Uninfected vs KRV+Propionic | 0.142 | 0.258 |
|  |  | KRV+Butyric vs Butyric | 0.29 | 0.443 |
|  |  | KRV+Formic vs Formic | 0.29 | 0.443 |
|  |  | KRV+Propionic vs Propionic | 0.29 | 0.443 |
|  |  | KRV vs Butyric | 0.367 | 0.528 |
|  |  | KRV vs Formic | 0.367 | 0.528 |
|  |  | KRV vs Propionic | 0.367 | 0.528 |
| Firmicutes | 0.008 | KRV vs Butyric | 0.005 | 0.021 |
|  |  | KRV vs Formic | 0.005 | 0.021 |
|  |  | KRV vs Propionic | 0.005 | 0.021 |
|  |  | Uninfected vs Butyric | 0.007 | 0.027 |
|  |  | Uninfected vs Formic | 0.007 | 0.027 |
|  |  | Uninfected vs Propionic | 0.007 | 0.027 |
|  |  | KRV+Butyric vs Butyric | 0.009 | 0.034 |
|  |  | KRV+Formic vs Formic | 0.009 | 0.034 |
|  |  | KRV+Propionic vs Propionic | 0.009 | 0.034 |
|  |  | Uninfected vs KRV | 0.851 | 0.988 |
|  |  | Uninfected vs KRV+Butyric | 0.904 | 1 |
|  |  | Uninfected vs KRV+Formic | 0.904 | 1 |
|  |  | Uninfected vs KRV+Propionic | 0.904 | 1 |
|  |  | KRV vs KRV+Butyric | 0.962 | 1 |
|  |  | KRV vs KRV+Formic | 0.962 | 1 |
|  |  | KRV vs KRV+Propionic | 0.962 | 1 |
| 4C0d-2 | 0.008 | Uninfected vs Butyric | 0.002 | 0.012 |
|  |  | Uninfected vs Formic | 0.002 | 0.012 |
|  |  | Uninfected vs Propionic | 0.002 | 0.012 |
|  |  | Uninfected vs KRV | 0.066 | 0.142 |
|  |  | Uninfected vs KRV+Butyric | 0.069 | 0.147 |
|  |  | Uninfected vs KRV+Formic | 0.069 | 0.147 |
|  |  | Uninfected vs KRV+Propionic | 0.069 | 0.147 |
|  |  | KRV vs Butyric | 0.085 | 0.174 |
|  |  | KRV vs Formic | 0.085 | 0.174 |
|  |  | KRV vs Propionic | 0.085 | 0.174 |
|  |  | KRV+Butyric vs Butyric | 0.146 | 0.26 |
|  |  | KRV+Formic vs Formic | 0.146 | 0.26 |
|  |  | KRV+Propionic vs Propionic | 0.146 | 0.26 |
|  |  | KRV vs KRV+Butyric | 0.857 | 0.992 |
|  |  | KRV vs KRV+Formic | 0.857 | 0.992 |
|  |  | KRV vs KRV+Propionic | 0.857 | 0.992 |
| Streptococcaceae | 0.008 | Uninfected vs Butyric | 0.005 | 0.023 |
|  |  | Uninfected vs Formic | 0.005 | 0.023 |
|  |  | Uninfected vs Propionic | 0.005 | 0.023 |
|  |  | KRV vs Butyric | 0.014 | 0.043 |
|  |  | KRV vs Formic | 0.014 | 0.043 |
|  |  | KRV vs Propionic | 0.014 | 0.043 |
|  |  | Uninfected vs KRV+Butyric | 0.03 | 0.078 |
|  |  | Uninfected vs KRV+Formic | 0.03 | 0.078 |
|  |  | Uninfected vs KRV+Propionic | 0.03 | 0.078 |
|  |  | KRV vs KRV+Butyric | 0.074 | 0.156 |
|  |  | KRV vs KRV+Formic | 0.074 | 0.156 |
|  |  | KRV vs KRV+Propionic | 0.074 | 0.156 |
|  |  | KRV+Butyric vs Butyric | 0.403 | 0.562 |
|  |  | KRV+Formic vs Formic | 0.403 | 0.562 |
|  |  | KRV+Propionic vs Propionic | 0.403 | 0.562 |
|  |  | Uninfected vs KRV | 0.641 | 0.784 |
| mitochondria | 0.009 | KRV+Butyric vs Butyric | 0.002 | 0.011 |
|  |  | KRV+Formic vs Formic | 0.002 | 0.011 |
|  |  | KRV+Propionic vs Propionic | 0.002 | 0.011 |
|  |  | KRV vs KRV+Butyric | 0.002 | 0.014 |
|  |  | KRV vs KRV+Formic | 0.002 | 0.014 |
|  |  | KRV vs KRV+Propionic | 0.002 | 0.014 |
|  |  | Uninfected vs KRV+Butyric | 0.004 | 0.019 |
|  |  | Uninfected vs KRV+Formic | 0.004 | 0.019 |
|  |  | Uninfected vs KRV+Propionic | 0.004 | 0.019 |
|  |  | Uninfected vs Butyric | 0.406 | 0.562 |
|  |  | Uninfected vs Formic | 0.406 | 0.562 |
|  |  | Uninfected vs Propionic | 0.406 | 0.562 |
|  |  | KRV vs Butyric | 0.492 | 0.652 |
|  |  | KRV vs Formic | 0.492 | 0.652 |
|  |  | KRV vs Propionic | 0.492 | 0.652 |
|  |  | Uninfected vs KRV | 0.859 | 0.993 |
| Enhydrobacter | 0.012 | Uninfected vs KRV | 0.082 | 0.172 |
|  |  | KRV vs KRV+Butyric | 0.119 | 0.227 |
|  |  | KRV vs KRV+Formic | 0.119 | 0.227 |
|  |  | KRV vs KRV+Propionic | 0.119 | 0.227 |
|  |  | KRV vs Butyric | 0.153 | 0.267 |
|  |  | KRV vs Formic | 0.153 | 0.267 |
|  |  | KRV vs Propionic | 0.153 | 0.267 |
|  |  | Uninfected vs KRV+Butyric | 1 | 1 |
|  |  | Uninfected vs Butyric | 1 | 1 |
|  |  | KRV+Butyric vs Butyric | 1 | 1 |
|  |  | Uninfected vs KRV+Formic | 1 | 1 |
|  |  | Uninfected vs Formic | 1 | 1 |
|  |  | KRV+Formic vs Formic | 1 | 1 |
|  |  | Uninfected vs KRV+Propionic | 1 | 1 |
|  |  | Uninfected vs Propionic | 1 | 1 |
|  |  | KRV+Propionic vs Propionic | 1 | 1 |
| Delftia | 0.016 | Uninfected vs KRV | 0.066 | 0.142 |
|  |  | KRV vs KRV+Butyric | 0.098 | 0.198 |
|  |  | KRV vs KRV+Formic | 0.098 | 0.198 |
|  |  | KRV vs KRV+Propionic | 0.098 | 0.198 |
|  |  | KRV vs Butyric | 0.13 | 0.241 |
|  |  | KRV vs Formic | 0.13 | 0.241 |
|  |  | KRV vs Propionic | 0.13 | 0.241 |
|  |  | Uninfected vs KRV+Butyric | 1 | 1 |
|  |  | Uninfected vs Butyric | 1 | 1 |
|  |  | KRV+Butyric vs Butyric | 1 | 1 |
|  |  | Uninfected vs KRV+Formic | 1 | 1 |
|  |  | Uninfected vs Formic | 1 | 1 |
|  |  | KRV+Formic vs Formic | 1 | 1 |
|  |  | Uninfected vs KRV+Propionic | 1 | 1 |
|  |  | Uninfected vs Propionic | 1 | 1 |
|  |  | KRV+Propionic vs Propionic | 1 | 1 |
| Coprococcus | 0.017 | KRV vs KRV+Butyric | 0.027 | 0.074 |
|  |  | KRV vs KRV+Formic | 0.027 | 0.074 |
|  |  | KRV vs KRV+Propionic | 0.027 | 0.074 |
|  |  | KRV vs Butyric | 0.17 | 0.285 |
|  |  | KRV vs Formic | 0.17 | 0.285 |
|  |  | KRV vs Propionic | 0.17 | 0.285 |
|  |  | Uninfected vs KRV+Butyric | 0.219 | 0.352 |
|  |  | Uninfected vs KRV+Formic | 0.219 | 0.352 |
|  |  | Uninfected vs KRV+Propionic | 0.219 | 0.352 |
|  |  | Uninfected vs KRV | 0.243 | 0.381 |
|  |  | KRV+Butyric vs Butyric | 0.515 | 0.67 |
|  |  | KRV+Formic vs Formic | 0.515 | 0.67 |
|  |  | KRV+Propionic vs Propionic | 0.515 | 0.67 |
|  |  | Uninfected vs Butyric | 0.669 | 0.805 |
|  |  | Uninfected vs Formic | 0.669 | 0.805 |
|  |  | Uninfected vs Propionic | 0.669 | 0.805 |
| Allobaculum | 0.019 | Uninfected vs KRV+Butyric | 0.003 | 0.017 |
|  |  | Uninfected vs KRV+Formic | 0.003 | 0.017 |
|  |  | Uninfected vs KRV+Propionic | 0.003 | 0.017 |
|  |  | Uninfected vs KRV | 0.004 | 0.021 |
|  |  | KRV+Butyric vs Butyric | 0.01 | 0.036 |
|  |  | KRV+Formic vs Formic | 0.01 | 0.036 |
|  |  | KRV+Propionic vs Propionic | 0.01 | 0.036 |
|  |  | KRV vs Butyric | 0.016 | 0.048 |
|  |  | KRV vs Formic | 0.016 | 0.048 |
|  |  | KRV vs Propionic | 0.016 | 0.048 |
|  |  | KRV vs KRV+Butyric | 0.666 | 0.805 |
|  |  | KRV vs KRV+Formic | 0.666 | 0.805 |
|  |  | KRV vs KRV+Propionic | 0.666 | 0.805 |
|  |  | Uninfected vs Butyric | 0.966 | 1 |
|  |  | Uninfected vs Formic | 0.966 | 1 |
|  |  | Uninfected vs Propionic | 0.966 | 1 |
| RC9-gut-group | 0.022 | KRV+Butyric vs Butyric | 0.039 | 0.097 |
|  |  | KRV+Formic vs Formic | 0.039 | 0.097 |
|  |  | KRV+Propionic vs Propionic | 0.039 | 0.097 |
|  |  | KRV vs Butyric | 0.063 | 0.14 |
|  |  | KRV vs Formic | 0.063 | 0.14 |
|  |  | KRV vs Propionic | 0.063 | 0.14 |
|  |  | Uninfected vs Butyric | 0.139 | 0.254 |
|  |  | Uninfected vs Formic | 0.139 | 0.254 |
|  |  | Uninfected vs Propionic | 0.139 | 0.254 |
|  |  | Uninfected vs KRV+Butyric | 0.387 | 0.548 |
|  |  | Uninfected vs KRV+Formic | 0.387 | 0.548 |
|  |  | Uninfected vs KRV+Propionic | 0.387 | 0.548 |
|  |  | Uninfected vs KRV | 0.623 | 0.768 |
|  |  | KRV vs KRV+Butyric | 0.668 | 0.805 |
|  |  | KRV vs KRV+Formic | 0.668 | 0.805 |
|  |  | KRV vs KRV+Propionic | 0.668 | 0.805 |
| Peptostreptococcaceae | 0.023 | Uninfected vs KRV+Butyric | 0.005 | 0.023 |
|  |  | Uninfected vs KRV+Formic | 0.005 | 0.023 |
|  |  | Uninfected vs KRV+Propionic | 0.005 | 0.023 |
|  |  | KRV vs KRV+Butyric | 0.017 | 0.049 |
|  |  | KRV vs KRV+Formic | 0.017 | 0.049 |
|  |  | KRV vs KRV+Propionic | 0.017 | 0.049 |
|  |  | Uninfected vs Butyric | 0.064 | 0.14 |
|  |  | Uninfected vs Formic | 0.064 | 0.14 |
|  |  | Uninfected vs Propionic | 0.064 | 0.14 |
|  |  | KRV vs Butyric | 0.145 | 0.26 |
|  |  | KRV vs Formic | 0.145 | 0.26 |
|  |  | KRV vs Propionic | 0.145 | 0.26 |
|  |  | KRV+Butyric vs Butyric | 0.459 | 0.618 |
|  |  | KRV+Formic vs Formic | 0.459 | 0.618 |
|  |  | KRV+Propionic vs Propionic | 0.459 | 0.618 |
|  |  | Uninfected vs KRV | 0.605 | 0.758 |
| Bacilli | 0.023 | Uninfected vs Butyric | 0.007 | 0.028 |
|  |  | Uninfected vs Formic | 0.007 | 0.028 |
|  |  | Uninfected vs Propionic | 0.007 | 0.028 |
|  |  | Uninfected vs KRV | 0.013 | 0.04 |
|  |  | KRV+Butyric vs Butyric | 0.063 | 0.14 |
|  |  | KRV+Formic vs Formic | 0.063 | 0.14 |
|  |  | KRV+Propionic vs Propionic | 0.063 | 0.14 |
|  |  | KRV vs KRV+Butyric | 0.147 | 0.26 |
|  |  | KRV vs KRV+Formic | 0.147 | 0.26 |
|  |  | KRV vs KRV+Propionic | 0.147 | 0.26 |
|  |  | Uninfected vs KRV+Butyric | 0.391 | 0.55 |
|  |  | Uninfected vs KRV+Formic | 0.391 | 0.55 |
|  |  | Uninfected vs KRV+Propionic | 0.391 | 0.55 |
|  |  | KRV vs Butyric | 0.479 | 0.638 |
|  |  | KRV vs Formic | 0.479 | 0.638 |
|  |  | KRV vs Propionic | 0.479 | 0.638 |
| Prevotellaceae | 0.029 | KRV vs Butyric | 0.166 | 0.283 |
|  |  | KRV vs Formic | 0.166 | 0.283 |
|  |  | KRV vs Propionic | 0.166 | 0.283 |
|  |  | Uninfected vs KRV | 0.308 | 0.466 |
|  |  | KRV+Butyric vs Butyric | 0.359 | 0.525 |
|  |  | KRV+Formic vs Formic | 0.359 | 0.525 |
|  |  | KRV+Propionic vs Propionic | 0.359 | 0.525 |
|  |  | Uninfected vs Butyric | 0.572 | 0.732 |
|  |  | Uninfected vs Formic | 0.572 | 0.732 |
|  |  | Uninfected vs Propionic | 0.572 | 0.732 |
|  |  | Uninfected vs KRV+Butyric | 0.638 | 0.782 |
|  |  | Uninfected vs KRV+Formic | 0.638 | 0.782 |
|  |  | Uninfected vs KRV+Propionic | 0.638 | 0.782 |
|  |  | KRV vs KRV+Butyric | 0.655 | 0.796 |
|  |  | KRV vs KRV+Formic | 0.655 | 0.796 |
|  |  | KRV vs KRV+Propionic | 0.655 | 0.796 |
| Papillibacter | 0.033 | Uninfected vs Butyric | 0.006 | 0.025 |
|  |  | Uninfected vs Formic | 0.006 | 0.025 |
|  |  | Uninfected vs Propionic | 0.006 | 0.025 |
|  |  | KRV vs Butyric | 0.007 | 0.028 |
|  |  | KRV vs Formic | 0.007 | 0.028 |
|  |  | KRV vs Propionic | 0.007 | 0.028 |
|  |  | KRV+Butyric vs Butyric | 0.011 | 0.038 |
|  |  | KRV+Formic vs Formic | 0.011 | 0.038 |
|  |  | KRV+Propionic vs Propionic | 0.011 | 0.038 |
|  |  | Uninfected vs KRV | 0.919 | 1 |
|  |  | Uninfected vs KRV+Butyric | 0.964 | 1 |
|  |  | KRV vs KRV+Butyric | 0.964 | 1 |
|  |  | Uninfected vs KRV+Formic | 0.964 | 1 |
|  |  | KRV vs KRV+Formic | 0.964 | 1 |
|  |  | Uninfected vs KRV+Propionic | 0.964 | 1 |
|  |  | KRV vs KRV+Propionic | 0.964 | 1 |
| Propionibacteriu | 0.035 | Uninfected vs KRV | 0.017 | 0.051 |
|  |  | KRV vs KRV+Butyric | 0.032 | 0.082 |
|  |  | KRV vs KRV+Formic | 0.032 | 0.082 |
|  |  | KRV vs KRV+Propionic | 0.032 | 0.082 |
|  |  | KRV vs Butyric | 0.049 | 0.114 |
|  |  | KRV vs Formic | 0.049 | 0.114 |
|  |  | KRV vs Propionic | 0.049 | 0.114 |
|  |  | Uninfected vs KRV+Butyric | 1 | 1 |
|  |  | Uninfected vs Butyric | 1 | 1 |
|  |  | KRV+Butyric vs Butyric | 1 | 1 |
|  |  | Uninfected vs KRV+Formic | 1 | 1 |
|  |  | Uninfected vs Formic | 1 | 1 |
|  |  | KRV+Formic vs Formic | 1 | 1 |
|  |  | Uninfected vs KRV+Propionic | 1 | 1 |
|  |  | Uninfected vs Propionic | 1 | 1 |
|  |  | KRV+Propionic vs Propionic | 1 | 1 |
| Clostridiales | 0.036 | Uninfected vs KRV+Butyric | 0.079 | 0.164 |
|  |  | Uninfected vs KRV+Formic | 0.079 | 0.164 |
|  |  | Uninfected vs KRV+Propionic | 0.079 | 0.164 |
|  |  | KRV+Butyric vs Butyric | 0.117 | 0.224 |
|  |  | KRV+Formic vs Formic | 0.117 | 0.224 |
|  |  | KRV+Propionic vs Propionic | 0.117 | 0.224 |
|  |  | Uninfected vs KRV | 0.324 | 0.488 |
|  |  | KRV vs KRV+Butyric | 0.365 | 0.528 |
|  |  | KRV vs KRV+Formic | 0.365 | 0.528 |
|  |  | KRV vs KRV+Propionic | 0.365 | 0.528 |
|  |  | KRV vs Butyric | 0.374 | 0.532 |
|  |  | KRV vs Formic | 0.374 | 0.532 |
|  |  | KRV vs Propionic | 0.374 | 0.532 |
|  |  | Uninfected vs Butyric | 0.934 | 1 |
|  |  | Uninfected vs Formic | 0.934 | 1 |
|  |  | Uninfected vs Propionic | 0.934 | 1 |
| Mucispirillum | 0.043 | KRV vs KRV+Butyric | 0.004 | 0.019 |
|  |  | KRV vs KRV+Formic | 0.004 | 0.019 |
|  |  | KRV vs KRV+Propionic | 0.004 | 0.019 |
|  |  | Uninfected vs KRV+Butyric | 0.039 | 0.097 |
|  |  | Uninfected vs KRV+Formic | 0.039 | 0.097 |
|  |  | Uninfected vs KRV+Propionic | 0.039 | 0.097 |
|  |  | KRV vs Butyric | 0.076 | 0.159 |
|  |  | KRV vs Formic | 0.076 | 0.159 |
|  |  | KRV vs Propionic | 0.076 | 0.159 |
|  |  | Uninfected vs KRV | 0.292 | 0.445 |
|  |  | Uninfected vs Butyric | 0.345 | 0.507 |
|  |  | Uninfected vs Formic | 0.345 | 0.507 |
|  |  | Uninfected vs Propionic | 0.345 | 0.507 |
|  |  | KRV+Butyric vs Butyric | 0.362 | 0.526 |
|  |  | KRV+Formic vs Formic | 0.362 | 0.526 |
|  |  | KRV+Propionic vs Propionic | 0.362 | 0.526 |

*Highlighted are adjusted FDR *p*-values <0.1
