# Supplementary figures and images for: Maternal treatment with short-chain fatty acids modulates the intestinal microbiota and immunity and ameliorates type 1 diabetes in the offspring
Source: PLoS One. 2017 Sep 8;12(9):e0183786. doi: 10.1371/journal.pone.0183786 (PMC5590848; doi:10.1371/journal.pone.0183786)

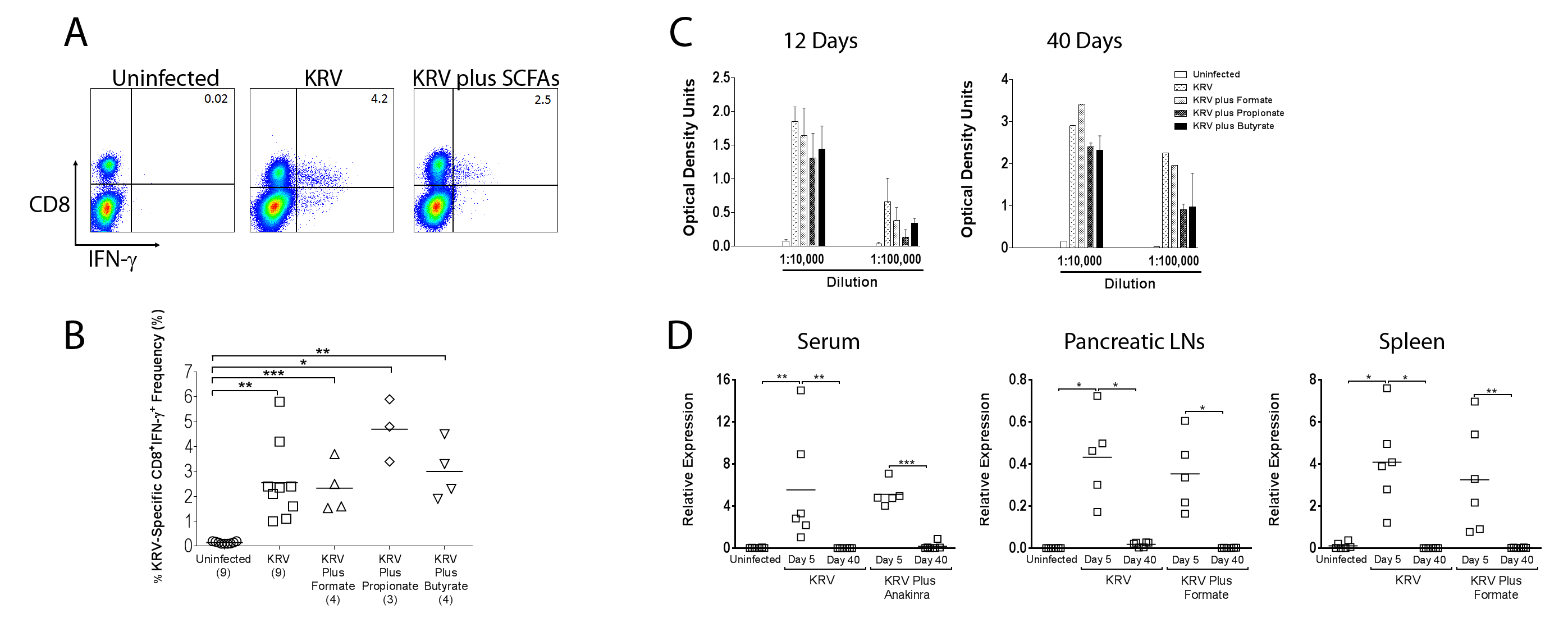
**S1 Fig. KRV-specific adaptive immunity and virus clearance in rats treated with SCFAs**

Supplement: S1 Fig — Rats were either left untreated, were injected with KRV, or were administered with KRV plus SCFA using treatment protocol 1. Panels A and B: Spleen cells were harvested on day 12 following infection and activated in the presence of KRV (n = 3–9 per group). Representative flow cytometry images and frequencies of IFN-γ+ cells out of the total CD8+ cells are shown in Panels A and B, respectively. The horizontal (IFN-γ) and vertical (CD8) axes indicate the fluorescence intensity. The proportion of CD8+ cells out of the total IFN-γ+ cells is shown in the upper right quadrant of each flow panel. Panels C: Blood samples were removed on days 12 and 40 (n = 2–3 per group). The serum was diluted as indicated in the figure and was assayed for the presence of virus-specific Abs. Each bar represents the mean value. Panel D: Blood, spleens, and pancreatic LNs were removed 5 and 40 days after infection ((n = 5–6 per group). RNA was extracted, and the level of KRV transcripts was determined by quantitative RT-PCR. The results are expressed as the expression of the gene mRNA relative to the expression of β-actin. Statistical analyses were performed using an ANOVA with Bonferroni's multiple comparison adjustments. *p < 0.001; **p < 0.01; ***p < 0.05. (DOCX) [file pone.0183786.s003.docx]
